# Global Patterns of Subgenome Evolution in Organelle-Targeted Genes of Six Allotetraploid Angiosperms

**DOI:** 10.1093/molbev/msac074

**Published:** 2022-04-06

**Authors:** Joel Sharbrough, Justin L. Conover, Matheus Fernandes Gyorfy, Corrinne E. Grover, Emma R. Miller, Jonathan F. Wendel, Daniel B. Sloan

**Affiliations:** 1 Department of Biology, Colorado State University, Fort Collins, CO, USA; 2 Department of Biology, New Mexico Institute of Mining and Technology, Socorro, NM, USA; 3 Department of Ecology, Evolution, and Organismal Biology, Iowa State University, Ames, IA, USA

**Keywords:** allopolyploidy, *Brachypodium hybridum*, *Chenopodium quinoa*, chloroplast, *Coffea arabica*, CyMIRA, cytonuclear incompatibility, *Gossypium hirsutum*, mitochondrion, *Nicotiana tabacum*, *Triticum dicoccoides*

## Abstract

Whole-genome duplications (WGDs) are a prominent process of diversification in eukaryotes. The genetic and evolutionary forces that WGD imposes on cytoplasmic genomes are not well understood, despite the central role that cytonuclear interactions play in eukaryotic function and fitness. Cellular respiration and photosynthesis depend on successful interaction between the 3,000+ nuclear-encoded proteins destined for the mitochondria or plastids and the gene products of cytoplasmic genomes in multi-subunit complexes such as OXPHOS, organellar ribosomes, Photosystems I and II, and Rubisco. Allopolyploids are thus faced with the critical task of coordinating interactions between the nuclear and cytoplasmic genes that were inherited from different species. Because the cytoplasmic genomes share a more recent history of common descent with the maternal nuclear subgenome than the paternal subgenome, evolutionary “mismatches” between the paternal subgenome and the cytoplasmic genomes in allopolyploids might lead to the accelerated rates of evolution in the paternal homoeologs of allopolyploids, either through relaxed purifying selection or strong directional selection to rectify these mismatches. We report evidence from six independently formed allotetraploids that the subgenomes exhibit unequal rates of protein-sequence evolution, but we found no evidence that cytonuclear incompatibilities result in altered evolutionary trajectories of the paternal homoeologs of organelle-targeted genes. The analyses of gene content revealed mixed evidence for whether the organelle-targeted genes are lost more rapidly than the non-organelle-targeted genes. Together, these global analyses provide insights into the complex evolutionary dynamics of allopolyploids, showing that the allopolyploid subgenomes have separate evolutionary trajectories despite sharing the same nucleus, generation time, and ecological context.

## Introduction

Whole-genome duplication (WGD) events, in which the nuclear genome is doubled via polyploidization, are among the most profound mutational changes observed in nature. The high frequency of WGDs, especially among flowering plants (Jiao et al. 2011; Wendel 2015; Ruprecht et al. 2017; One Thousand Plant Transcriptomes Initiative 2019), makes them a major force in genome evolution. Accordingly, evolutionary biologists have had a great deal of interest in exploring the consequences of and responses to WGD. The ensuing studies have shown that the effects of WGDs are far-ranging, including the silencing and loss of duplicated genes (Anssour et al. 2009; Schnable et al. 2011; Buggs et al. 2012; Liu et al. 2014; Mirzaghaderi and Mason 2017; Cheng et al. 2018; Wendel et al. 2018), mobilization of previously dormant transposable elements (Petit et al. 2010; Gao et al. 2016; Senerchia et al. 2016; Springer et al. 2016; Vicient and Casacuberta 2017; Nieto Feliner et al. 2020), intergenomic gene conversion and homoeologous chromosome exchanges (Chester et al. 2012; Chalhoub et al. 2014; Guo et al. 2014; Jarvis et al. 2017; Chen et al. 2018; Bertioli et al. 2019; Li et al. 2019b; Mason and Wendel 2020), alterations of epigenetic marks (Madlung et al. 2002; Salmon et al. 2005; Shcherban et al. 2008; Fulneček et al. 2009; Akagi et al. 2016; Chen et al. 2017a; Song et al. 2017; Ding and Chen 2018), massive, genome-wide transcriptional rewiring (Schnable et al. 2011; Combes et al. 2013; Akama et al. 2014; Hu et al. 2016; Yang et al. 2016; Edger et al. 2017; Ramírez-González et al. 2018; Oberprieler et al. 2019; Landis et al. 2020), and a host of other associated physiological, ecological, and life-history changes (Stebbins 1940; Levin 1983; Otto and Whitton 2000; Ramsey and Schemske 2003; Otto 2007; Leitch and Leitch 2008; Van de Peer et al. 2009; Madlung 2013; Soltis et al. 2014; Yang et al. 2018; Doyle and Coate 2019; Bomblies 2020; Fox et al. 2020). Whole-genome duplications are also expected to produce novel interactions between the nuclear genome and the mitochondrial and plastid genomes (Sharbrough et al. 2017), but this dimension of allopolyploid evolution has received relatively little attention (but see Gong et al. 2012, 2014; Sehrish et al. 2015; Wang et al. 2017; Ferreira de Carvalho et al. 2019; Zhai et al. 2019; Li et al. 2020).

Cytonuclear interactions are themselves the result of gene transfers from the cytoplasmic genomes (mitochondrial and plastid) to the nuclear genome or the recruitment of existing nuclear-encoded proteins to function in these organelles (Kleine et al. 2009; Sloan et al. 2018). As a result, the vast majority of the ∼2,000 proteins that comprise the mitochondrial proteome (Millar 2007) and ∼3,000 proteins that comprise the plastid proteome (van Wijk and Baginsky 2011) are nuclear-encoded (Forsythe et al. 2019). Many of these nuclear-encoded proteins directly interact with gene products from the cytoplasmic genomes to form heteromeric complexes (e.g., Rubisco, Photosystems I and II, organellar ribosomes, and the enzymes that comprise the mitochondrial electron transport chain). Additionally, the replication, expression, and posttranscriptional modifications of cytoplasmic genomes are dependent on nuclear-encoded proteins (Day and Madesis 2007; Cupp and Nielsen 2014; Gualberto and Newton 2017; Morley et al. 2019), as are the many biosynthetic and signaling functions of the mitochondria and plastids (Woodson and Chory 2008; Liere et al. 2011; Weihe et al. 2012; Chan et al. 2016; Huang et al. 2016; Richardson et al. 2017; Krupinska et al. 2020). Taken together, the cellular and metabolic functions that result from cytonuclear interactions, especially aerobic respiration and photosynthesis, are critically important to eukaryotic health and fitness (Pike et al. 2007; Barreto and Burton 2013; Dowling 2014; Kremnev and Strand 2014; Hill et al. 2019). Perturbations to one genomic compartment can, therefore, have dramatic consequences for the other genomic compartments (Rand et al. 2004; Weng et al. 2016; Havird et al. 2017; Barreto et al. 2018; Li et al. 2019a; Yan et al. 2019; Hill 2020), so much so that incompatibilities between the nuclear and cytoplasmic genomes may be a potent force in generating and reinforcing species boundaries (Mayr 1986; Breeuwer and Werren 1993; Chapman and Mulcahy 1997; Turelli and Moyle 2007; Gershoni et al. 2009; Greiner et al. 2011; Burton and Barreto 2012; Hill 2016; Sloan et al. 2017; Postel and Touzet 2020).

Allopolyploidization, a WGD event resulting from a genome merger of two differentiated species (Grant 1981; Wendel and Doyle 2005; Doyle and Sherman-Broyles 2017), is expected to perturb cytonuclear interactions because the cytoplasmic genomes have a more recent history of shared descent with one nuclear subgenome than the other (Sharbrough et al. 2017). Researchers have hypothesized several immediate and evolutionary responses that may mitigate any resulting deleterious consequences. First, maternally biased nuclear gene expression in recently formed allopolyploid lineages could alleviate the deleterious consequences of incompatibilities between the paternal nuclear subgenome and cytoplasmic genomes (Gong et al. 2012). Over time, evolutionary rates may vary across the nuclear subgenomes, with paternal copies of the organelle-targeted genes evolving faster than maternal copies, either as a reflection of relaxed selection (Wertheim et al. 2015) or positive selection to rectify mismatches with the cytoplasmic genomes (Hill 2020). In the long run, the paternal copies of organelle-targeted genes may be altered more frequently than the maternal copies as a result of maternally biased gene conversion (Gong et al. 2014; Li et al. 2020), homoeologous exchange (Mason and Wendel 2020), or complete excision from the genome via pseudogenization and gene loss (Sehrish et al. 2015).

Prior to the 21st century, relatively little attention was paid to how allopolyploidization *per se* affected cytonuclear interactions (reviewed in Wendel (2000)). However, much was already beginning to emerge about the importance of cytonuclear interactions for plant development (Taylor 1989; Leon et al. 1998) and the molecular causes and consequences of cytoplasmic male sterility of the allopolyploid crops (Kück and Wricke 1995), in which maternally inherited factors, often the mitochondria, combine to produce nonfunctional pollen (Schnable and Wise 1998). Indeed, cytoplasmically male sterile hexaploid wheat was first reported in 1966 (Chauhan and Singh 1966), as well as in many other economically important allopolyploids (reviewed in Chen and Liu (2014)). An earlier study in allotetraploid *Brassica napus* revealed that separate nuclear restorers ameliorated cytoplasmic male sterility caused by the mitochondrial genome (Fang and McVetty 1989), which was speculated to have resulted from separate subgenomes (Singh and Brown 1991). Later studies confirmed this finding, showing that the nuclear-encoded pentatricopeptide repeat (PPR)-containing genes restored male function by regulating mitochondrial transcripts (Singh et al. 1996; Li et al. 1998; Bentolila et al. 2002). These powerful genetic dissections notwithstanding, understanding the role of genome mergers on cytonuclear molecular coevolution was not practical until the advent of remarkable polyploid genome assemblies whose subgenomes were successfully separated by long reads and optical mapping.

More recent investigations into the predicted outcomes of cytonuclear incompatibilities in allopolyploids have so far had mixed results. Rubisco, in particular, has been a primary focus as the nuclear-encoded small subunit *rbcS* appears to have undergone maternally biased gene conversion and exhibit maternally biased gene expression in some allopolyploids, such as cotton, tobacco, *Arabidopsis suecica*, peanut, and wheat (Gong et al. 2012, 2014; Li et al. 2020). Synthetic and recently formed allopolyploids show more inconsistent support. For example, *Tragopogon miscellus* exhibits maternally biased expression of *rbcS*, while its reciprocally formed congener *Tragopogon mirus* does not (Sehrish et al. 2015). Synthetic allotetraploid rice showed little evidence of the maternally biased expression of *rbcS* (Wang et al. 2017), and synthetic allopolyploid *Cucumis × hytivus* displayed paternally biased expression of *rbcS* (Zhai et al. 2019). Generalizing the rules of cytonuclear biology from these handful of somewhat contradictory studies is made even more difficult because they all have considered a single cytonuclear complex only.

A more extensive survey of 110 nuclear genes encoding subunits involved in plastid protein complexes in allopolyploid *Brassica napus* did not find evidence for maternally biased expression or the retention of organelle-targeted genes (Ferreira de Carvalho et al. 2019). What remains to be evaluated is whether there are systematic rules that might explain the discrepancies among these earlier studies, and more generally, what the principles are that govern cytonuclear coevolution in plant allopolyploids. There are as yet no genome-wide investigations of the signatures of cytonuclear incompatibilities in a set of independently formed allopolyploids that differ both in terms of the amount of divergence between diploid progenitors (and therefore, the probability of cytonuclear incompatibilities; Maheshwari and Barbash 2011), or time since allopolyploidization (and therefore, the probability of an evolutionary response to cytonuclear incompatibilities; Song et al. 1995). The rapidly increasing availability of genome sequences for many allopolyploid genomes and their diploid relatives (e.g., *Brassica* [Wang et al. 2011; Chalhoub et al. 2014; Liu et al. 2014; Kioukis et al. 2020], cotton [Paterson et al. 2012; Chen et al. 2017b; Udall et al. 2019a, 2019b], wheat [Avni et al. 2017; Luo et al. 2017; Mascher et al. 2017; Ling et al. 2018; Zhu et al. 2019], peanut [Bertioli et al. 2016, 2019], coffee [Denoeud et al. 2014; Dereeper et al. 2015; Tran et al. 2018; Xu et al. 2020], tobacco [Sierro et al. 2013; Edwards et al. 2017], quinoa [Jarvis et al. 2017; Mangelson et al. 2019], and *Brachypodium* [International Brachypodium Initiative 2010; Gordon et al. 2020]) makes it possible to better understand the rules of cytonuclear biology in allopolyploid lineages.

Here, we evaluate the genome-wide patterns of molecular evolution in the organelle-targeted gene sets for six separate allotetraploid species: *Brachypodium hybridum*, *Chenopodium quinoa* (quinoa), *Coffea arabica* (coffee), *Gossypium hirsutum* (cotton), *Nicotiana tabacum* (tobacco), and *Triticum dicoccoides* (wild emmer wheat). We document the strong effects of subgenome on the overall rates and patterns of evolution, but find little evidence for the global signatures of cytonuclear incompatibilities across the polyploid systems. We also find that the organelle-targeted gene content is generally less biased across the subgenomes than the rest of the genome. Together, these genome-wide analyses of six independently formed allotetraploid species provide insights into the rules of polyploidy, a prominent process in eukaryotic diversification.

## Results

### Study Systems: Origins, Evolution, and Genomics of Six Allotetraploids

**Fig. 1.**
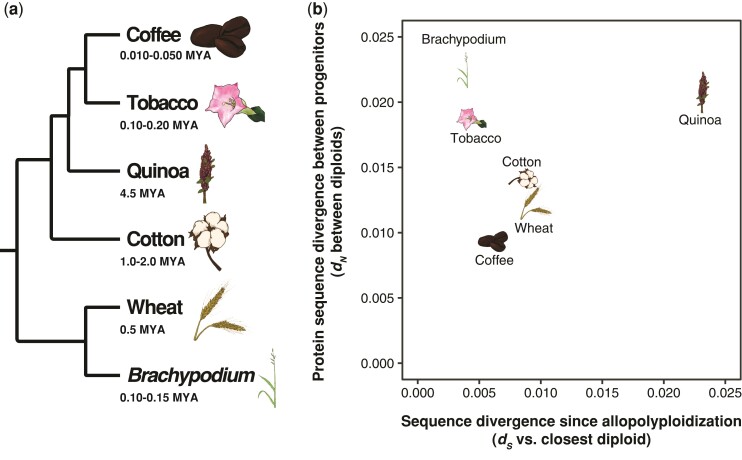
Evolutionary relationships and origins of six allotetraploid angiosperms. (*a*) Cladogram depicting evolutionary relationships among six independently derived allotetraploid angiosperms. (*b*) The scatter plot depicts the synonymous substitutions per synonymous site (*d_S_*) between the polyploid subgenome-diploid pair with the lowest amount of divergence on the *x*-axis as a proxy for the amount of time since allopolyploidization. Amino acid sequence divergence between subgenomes, measured as nonsynonymous substitutions per nonsynonymous site (*d_N_*) between the two diploid relatives, is shown on the *y*-axis. Higher levels of amino acid sequence divergence between the subgenomes increase the probability of a genetic incompatibility in the polyploid, whereas long periods since allopolyploidization increase the probability that evolutionary responses to incompatibilities are detectable in the polyploid.

Quinoa (*Chenopodium*, *Amaranthaceae*) represents the oldest allopolyploid in this study, having originated from a genome merger between the A (maternal) and B (paternal) *Chenopodium* lineages ∼4–6 Ma (Kolano et al. 2016; Jarvis et al. 2017; Zou et al. 2017). This allopolyploidization event, which gave rise to both *Ch. quinoa* and *Ch. berlandieri*, is thought to be distinct from that which gave rise to allohexaploid *Ch. album* (Krak et al. 2016). Tracing cytoplasmic donors has been tricky in quinoa primarily because the extant relatives bear so little resemblance to the ancient progenitors. Recent evidence from the plastid and mitochondrial genomes indicate that the A-genome species *Ch. watsonii* might be the best model of the maternal progenitor (Maughan et al. 2019), but *Ch. pallidicaule* is the only A lineage with a genome sequenced (Mangelson et al. 2019). There is some evidence of mixed inheritance of the cytoplasmic genomes in *Ch. album* (Gasquez et al. 1985), potentially indicating that the maternal progenitor might not be synonymous with the cytoplasmic donor; however, the A lineage is clearly the cytoplasmic lineage in this species (Maughan et al. 2019). Based on the estimates of *d_S_* between genomes, *Ch. suecicum*, the closest extant relative of the B lineage progenitor (*d_S_* between *Ch. suecicum* and *Ch. quinoa B* = 0.0233 synonymous substitutions per synonymous site, fig. 1*b*), appears to be a better model of the paternal origin than *Ch. pallidicaule* is for the maternal parent (*d_S_* between *Ch. pallidicaule* and *Ch. quinoaA* = 0.0316 synonymous substitution per synonymous site), although neither lineage is particularly closely related to the true diploid progenitors. There exists a substantial divergence between A and B lineages at the amino acid sequence level, providing a possibility for cytonuclear incompatibilities to arise (*d_N_* = 0.0206 nonsynonymous substitution per nonsynonymous site, fig. 1*b*).

The evolutionary history of allopolyploid cotton (*Gossypium*, *Malvaceae*) has been well characterized by Wendel (1989), Wendel et al. (2010), and Wendel and Grover (2015), with a primary allopolyploidization event occurring ∼1–2 Ma (Endrizzi et al. 1985; Wendel and Cronn 2003) between the A (maternal) and D (paternal) lineages. The AADD allotetraploid that resulted has since diversified into seven currently recognized allotetraploid species (Grover et al. 2012b; Wang et al. 2018; Hu et al. 2021). Mitochondrial (Chen et al. 2017a) and plastid genome (Wendel 1989) data indicate that the A lineage was the maternal progenitor (with genome representatives *G. arboreum* and *G. herbaceum*; Huang et al. 2020; Grover et al. 2021), and the D lineage was the paternal progenitor (with genome representative *G. raimondii*). Multiple polyploid genomes have been produced (Hu et al. 2019), with our focus here on allotetraploid *G. hirsutum*. Estimates of *d_S_* indicate that *G. arboreum* is a better model of the A subgenome progenitor (*d_S_* between *G. arboreum* and *G. hirsutumA* = 0.00908 synonymous substitutions per synonymous site, fig. 1*b*) compared with *G. raimondii* as a model of the D subgenome progenitor (*d_S_* between *G. raimondii* and *G. hirsutum D* = 0.0119 synonymous substitution per synonymous site). At the amino acid level, the A and D lineages differ by ∼1.33% (fig. 1*b*).

Allotetraploid wheat (*Triticum dicoccoides*, *Poaceae*) is the product of a genome merger between the A (*Triticum*) and B (*Aegilops*) lineages of the *Triticum/Aegilops* species complex, formed ∼0.5–1 Ma (Marcussen et al. 2014; Avni et al. 2017). Ancient cultivation of the tetraploid gave rise to *Triticum turgidum* (Maccaferri et al. 2019), and following fertilization by and subsequent genome merger with *Aegilops tauschii* (D genome, itself the product of a separate homoploid hybridization event between the A and B lineages (Li et al. 2015a, 2015b, 2019a; Sandve et al. 2015; Zhao et al. 2018)), to form the AABBDD hexaploid wheat *T. aestivum* (Marcussen et al. 2014; El Baidouri et al. 2017). Phylogenetic evidence from both organelles in tetraploid and hexaploid wheat supports the B lineage as the maternal progenitor (Graur et al. 1989; Gornicki et al. 2014); however, this lineage has not been discovered or has since gone extinct, and the more distantly related S lineage (represented by *A. speltoides*) is the closest known relative to the maternal progenitor for all polyploid wheat (Haider 2013). *Triticum urartu* serves as the primary genomic model for the A lineage and paternal progenitor (Ling et al. 2018). *Triticum urartu* is a substantially better model of the A subgenome progenitor (*d_S_* between *T. urartu* and *T. dicoccoides A* = 0.00991 synonymous substitutions per synonymous site, fig. 1*b*) than *A. speltoides* is of the B subgenome progenitor (*d_S_* = 0.0435 synonymous substitutions per synonymous site). There exists a similar degree of amino acid divergence between the A and B lineages as found between the cotton A and D lineages, with *d_N_* = 0.0131 nonsynonymous substitutions per nonsynonymous site (fig. 1*b*).

The tobacco genus *Nicotiana* (*Solanaceae*) is replete with polyploidy, ranging dramatically in age (Leitch et al. 2008). One such polyploid, *N. tabacum*, appears to have arisen <200,000 years ago in a merger between *N. sylvestris* and *N. tomentosiformis* (Murad et al. 2002; Knapp et al. 2004; Leitch et al. 2008). Although young, this merger represents the most divergence between diploid progenitors among the *Nicotiana* polyploids (Leitch et al. 2008). Plastid (Aoki and Ito 2000; Sasaki et al. 2003) and mitochondrial (Bland et al. 1985) data clearly establish *N. sylvestris* as the maternal donor, with a relatively little divergence between the progenitor and the extant species. Moreover, shared repeat sequences between the specific accessions of *N. tomentosiformis* and the *N. tabacum* T subgenome provide a positive evidence of *N. tomentosiformis* as the paternal progenitor (Murad et al. 2002). Both diploid taxa used in this study provide very good models of the diploid progenitors, with *N. sylvestris* being a slightly better model of the S genome (*d_S_* = 0.00448 synonymous substitutions per synonymous site, fig. 1*b*) than *N. tomentosiformis* is of the T subgenome (*d_S_* = 0.00736 synonymous substitutions per synonymous site). Still, the amino acid divergence between the S and T subgenomes is quite high (*d_N_* = 0.0200 nonsynonymous substitutions per nonsynonymous site, fig. 1*b*), making tobacco an excellent system in which to look for incompatibilities between the paternal T subgenome and the cytoplasmic genomes.

*Brachypodium hybridum* (*Poaceae*) is the product of an allotetraploidizaiton event between *B. stacei* (S lineage), and *B. distachyon* (D lineage), which has happened multiple times and in reciprocal directions (Gordon et al. 2020). The genome that is currently available for *B. hybridum* has a plastid genome most closely related to *B. stacei*, and appears to have formed ∼100,000–150,000 years ago (Gordon et al. 2020), but older polyploids also exist with the reciprocal maternal parentage (Gordon et al. 2017). Based on the apparent interchangeability between the D-lineage or S-lineage cytoplasms and the young age of the polyploid under consideration, we expected this species to be the least likely to exhibit biased patterns of cytonuclear evolution across the subgenomes. *Brachypodium stacei* is a very good model of the diploid progenitor of the S subgenome (*d_S_* = 0.00375 synonymous substitutions per synonymous site, fig. 1*b*), and *B. distachyon* is also a useful model of the *B. hybridum* D subgenome (*d_S_* = 0.00648 synonymous substitutions per synonymous site). The D and S lineages are the most diverged at the amino acid level of all polyploids considered here, with *d_N_* = 0.0224 nonsynonymous substitutions per nonsynonymous site (fig. 1*b*). Although *B. hybridum* is quite young, the degree of amino acid divergence provides a powerful system for detecting cytonuclear incompatibilities early, following polyploidization.

Allotetraploid coffee (*Coffea arabica*, *Rubiaceae*) was formed ∼10,000–50,000 years ago from a hybridization event between *Co. eugenioides* (E subgenome donor) and *Co. canephora* (C subgenome donor), with *Co. eugenioides* serving as the maternal progenitor (Cros et al. 1998; Simone et al. 2020). Both diploids represent good models of the polyploid progenitors, with *Co. eugenioides* being a slightly better model of the E subgenome (*d_S_* = 0.00623 synonymous substitutions per synonymous site, fig. 1*b*) than *Co. canephora* is of the C subgenome (*d_S_* = 0.00856 synonymous substitutions per synonymous site). The amino acid divergence between the E and C lineages is the lowest of all six polyploids (*d_N_* = 0.00934 nonsynonymous substitutions per nonsynonymous site, fig. 1*b*), indicating that cytonuclear incompatibilities may be less likely than in species with more divergent proteomes. This and the other allotetraploids together used in this study are further summarized in table 1 below and their divergence is described in fig. 1*b*.

**Table 1.** Summary of Allopolyploid Lineages Used in This Study.

Allopolyploid Species Complex	Time Since Polyploidization (Ma)	Subgenome Divergence (*d_S_*)^a^	Genome Type	Maternal Diploid (Genome Type)	Paternal Diploid (Genome Type)	Outgroup Species
*Brachypodium hybridum*	0.10–0.15	0.103	SSTT	*B. stacei* (SS)	*B. distachyon* (DD)	*Hordeum vulgare* (barley)
*Chenopodium quinoa* (quinoa)	4–6	0.105	AABB	*Ch. pallidicaule* (AA)	*Ch. suecicum* (BB)	*Spinacea oleracea* (spinach)
*Coffea* *arabica* (coffee)	0.01–0.05	0.026	EECC	*Co. eugenioides* (EE)	*Co. canephora* (CC)	*Gardenia jasminoides*
*Gossypium hirsutum* (cotton)	1–2	0.041	AADD	*G. arboreum* (AA)	*G. raimondii* (DD)	*Gossypioides kirkii*
*Nicotiana tabacum* (tobacco)	0.1–0.20	0.096	SSTT	*N. sylvestris* (SS)	*N. tomentosiformis* (SS)	*Solanum lycopersicum* (tomato)
*Triticum dicoccoides* (wheat)	0.5–1	0.076	BBAA	*A. speltoides* (SS)	*T. urartu* (AA)	*H. vulgare* (barley)

a Synonymous substitutions per synonymous site inferred from the concatenated estimates of *d_S_* from the non-organelle-targeted genes, see fig. 1.

### Orthologous Genes in Six Allopolyploid Species and Their Diploid Relatives

**Fig. 2.**
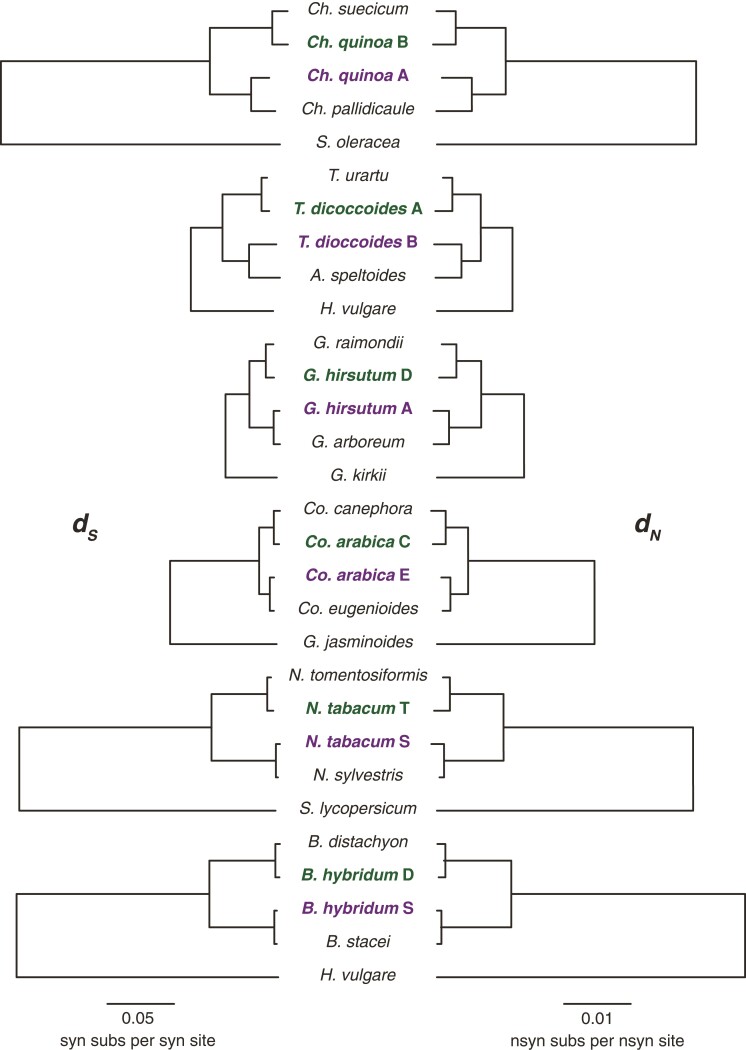
Synonymous and nonsynonymous rates of evolution in the genomes (and subgenomes) of focal allopolyploid systems. Substitution rates per site for synonymous (*d_S_*—left) and nonsynonymous (*d_N_*—right) sites from the concatenated analyses of non-organelle-targeted genes are represented by branch lengths for each genome (and subgenome). Allopolyploid systems are arranged from the oldest (top) to the youngest (bottom) from fig. 1*a*. The paternal subgenomes of allotetraploids are bolded in green (top subgenome) and the maternal subgenomes are bolded in purple (bottom subgenome).

To compare the rates and patterns of molecular evolution across the subgenomes of six allotetraploid angiosperms (fig. 1*a*), we inferred orthologous gene groups from the two polyploid subgenomes, the closest available diploid species for each subgenome, and an outgroup (fig. 2) using a combination of the phylogenetic and syntenic methods. The resulting orthologous gene groups are summarized in table 2, and additional details regarding their inference are provided in the Materials and Methods section as well as in supplementary fig. S1, Supplementary Material online.

**Table 2.** Orthologous Gene Groups in Six Allotetraploid Angiosperms.

Species	Phylogenetic Orthologous Groups	Syntenic Orthologous Groups	Merged, Single-copy Quintets^a^ (Phylo/Intersect/Synteny)	Filtered, Merged, Single-copy Quintets (Phylo/Intersect/Synteny)
Quinoa	10,511	17,896	(1,873/3,284/3,931)	(3,679/3,121/1,401)
Wheat	25,454	24,212	(3,070/3,602/3,508)	(1,754/1,759/691)
Cotton	29,504	31,841	(2,392/10,222/6,101)	(2,327/10,023/4,783)
Coffee	19,399	20,926	(2,005/3,869/789)	(1,225/2,379/428)
Tobacco	24,797	32,088	(8,837/166/56)	(8,536/163/52)
*Brachypodium*	24,854	34,440	(5,076/8,084/1,289)	(4,948/7,912/1,140)

a Single-copy quintets include orthologous gene groups with one and only one sequence from an outgroup, two closely related diploids, and two sequences from the allopolyploid.

The goal of our orthology inference methods was to produce orthologous “quintets,” containing one gene sequence each from the outgroup species and the two diploid model species and two gene sequences from the polyploid species, while also requiring that gene trees be consistent with the overall species tree. Both the syntenic and phylogenetic methods produced sizable numbers of identical quintets; however, there were many quintets only detectable using one method or the other. Tobacco was especially challenging for syntenic inference, as the relatively fragmented assemblies of the diploid *Nicotiana* reference genomes and the highly rearranged genome of allotetraploid *N. tabacum* made identifying syntenic blocks difficult. The largest syntenic block between any two of the genomes in this clade was only 57 genes long (*N. tabacum* and *Solanum lycopersicum*), and no syntenic block including *N. tomentosiformis* or *N. sylvestris* was longer than 22 genes. Quinoa highlighted a different issue that represents a common feature of polyploid genome assemblies in that many genes were located on contigs that are not anchored to chromosomes. Genes present in this fraction of the assembly can only be included in orthologous groups by phylogenetics, and they are often replete with repetitive elements, making it a likely spot for genome misassemblies (and subsequent errors in analyses that depend on them). Moreover, the quinoa genome contains cases of apparent homoeologous exchange in which genes were located on chromosomes from opposing subgenomes (see also Jarvis et al. 2017).

Variation in the assembly and annotation quality also represented a significant challenge in identifying the orthologous genes across genome assemblies produced by different groups with different underlying data. The most extreme example of this issue was the maternal diploid model for polyploid wheat, *A. speltoides*, which was represented only by a transcriptome assembly. Despite these and other hurdles, we were able to identify orthologous gene groups as well as the more strict group of single-copy quintets for each of these polyploid systems, which should present a useful resource for polyploid genomics moving forward. The *A. speltoides* transcriptome assembly, all the OrthoFinder results, phylogenetic gene trees with branch lengths, multi-species synteny networks, merged orthologous gene groups, CDS alignments, and the analyses of molecular evolution have been made available at https://doi.org/10.6084/m9.figshare.13473207. For the remainder of the manuscript, we report only on the results from the “Union” group of quintets that were identified by either phylogenetic or syntenic inference, but we have performed all the same analyses on the “Intersection” group, comprised only of those quintets that were identified by both methods, and have provided the results from those analyses in supplementary file S1, Supplementary Material online. Results obtained using the Intersection dataset did not substantively differ from those obtained using the Union dataset.

### Subgenomic Distributions of Organelle-Targeted Genes

To evaluate whether cytonuclear interactions affect subgenomic evolution in allopolyploid species, we first partitioned the genes by predicted subcellular targeting localization and cytonuclear interaction activity in each allopolyploid system. Cytonuclear interacting genes are defined here as those nuclear-encoded genes whose products interact with the mitochondrial/plastid genomes or gene products according to the Cytonuclear Molecular Interactions Reference for *Arabidopsis* (CyMIRA) database (Forsythe et al. 2019). CyMIRA indicates that the *Arabidopsis thaliana* nuclear genome has 1,773 genes that encode mitochondria-targeted products and 2,931 genes that encode plastid-targeted products. By propagating this classification across the six allotetraploids studied here, we found the means of 3,880 (SD = 730) genes with mitochondria-targeted products and 4,464 (SD = 731) genes with plastid-targeted products (table 3), which varies ∼60–70% among allotetraploid taxa. At least some of the observed variation among polyploids appears to be due to phylogeny, as the number of mitochondria-targeted genes and plastid-targeted genes varies extensively among diploids (25–30%, supplementary fig. S2, Supplementary Material online). Diploid relatives of our focal allotetraploids ranged from 17% fewer (*Chenopodium* diploids) to 108% more (*Nicotiana* diploids) mitochondria-targeted genes and from 37% fewer (*Triticum*, *Chenopodium* diploids) to 33% more (*Nicotiana* diploids) plastid-targeted genes than documented in *Arabidopsis* (supplementary fig. S2 and table S1, Supplementary Material online).

**Table 3.** Functional Gene Partitioning in Six Allotetraploid Angiosperms.

Species	Mitochondria-targeted	Mitochondria-targeted Interacting^a^	Mitochondria Enzyme Complexes^b^	Plastid-targeted	Plastid-targeted Interacting^a^	Plastid Enzyme Complexes^b^
Quinoa	2,830	894	279	3,528	686	215
Wheat	4,077	1,048	378	4,419	693	245
Cotton	4,728	1,232	458	5,670	800	307
Coffee	3,221	921	285	3,889	621	193
Tobacco	3,851	1,092	402	4,567	740	297
*Brachypodium*	4,540	981	339	4,684	674	238
Mean (SD)	3,880 (730)	1,031 (121)	358 (68)	4,464 (731)	704 (61)	250 (45)
*Arabidopsis thaliana* (diploid)	1,773	617	180	2,931	375	128

a Mitochondria- and plastid-targeted interacting genes are a subset of the total number of mitochondria- and plastid-targeted genes.

b Mitochondria and plastid enzyme complex genes are a subset of the total number of mitochondria- and plastid-targeted interacting genes.

Among the genes with mitochondria-targeted products, CyMIRA lists 617 *Arabidopsis thaliana* genes that have interactions with the mitochondrial genes or gene products and 180 genes with products that are directly involved in enzyme complexes with mitochondrially encoded subunits (i.e., mitoribosome, OXPHOS complexes, TAT complex). We expected to find roughly twice as many genes in each functional category for tetraploids as are present in *Arabidopsis*. In the six focal allotetraploids, we found that functional categories were increased 40–250% (per category/species) relative to *Arabidopsis thaliana*, with means of 1,031 (SD = 121) genes having interactions with mitochondrial genes or gene products and 358 (SD = 68) genes with products that are directly involved in those three mitochondrial enzyme complexes (MTECs). A similar pattern was observed for genes with plastid-targeted products. Where CyMIRA lists 375 *Arabidopsis thaliana* genes that have interactions with the plastid genes or gene products and 128 genes with products that are directly involved in enzyme complexes with plastid-encoded subunits (i.e., chlororibosome, Photosystems I and II, NDH, ATP synthase, Cytochrome b6f, Rubisco, Clp protease, ACCase), we found the means of 704 (SD = 61) and 250 (SD = 45) genes in the allotetraploids for those categories, respectively. Gene numbers for all the 55 functional gene categories and species are described in supplementary table S1, Supplementary Material online, gene IDs for each category and de novo targeting predictions are available at https://github.com/jsharbrough/CyMIRA_gene_classification/tree/master/Species_CyMIRA, and the physical distribution of organelle-targeted genes along polyploid chromosomes are shown in supplementary fig. S3, Supplementary Material online.

Polyploidization events are expected to perturb cytonuclear interactions in part because the cytoplasmic genomes suddenly exist inside a cell in which all of their nuclear-encoded interacting partners have been doubled. One possible evolutionary response to altered cytonuclear stoichiometry in the wake of WGD is that the nuclear-encoded organelle-targeted genes experience selection to rapidly return to a diploid-like state (De Smet et al. 2013; Li et al. 2016). We tested this hypothesis for both the mitochondria- and plastid-targeted nuclear genes in six independently formed allopolyploids using the combined diploid relatives as models for the ancestral allopolyploid state. We found that quinoa (*χ*^2^ = 54.40, *P* < 0.0001), wheat (χ^2^ = 660.23, *P* < 0.0001), tobacco (*χ*^2^ = 243.85, *P* < 0.0001), and *Brachypodium* (*χ*^2^ = 50.15, *P* < 0.0001) retain a significantly smaller proportion of the organelle-targeted genes in duplicate than the non-organelle-targeted genes, whereas, cotton (*χ*^2^ = 134.12, *P* < 0.0001) and coffee (*χ*^2^ = 13.40, *P* = 0.00025) exhibit the opposite pattern by retaining a significantly larger proportion of the organelle-targeted genes than the non-organelle-targeted genes (supplementary table S2, Supplementary Material online). Notably, excess retention of the organelle-targeted genes in cotton was also evident when we restricted our analysis to only include the subset of genes directly involved in mitochondrial (*χ*^2^ = 7.90, *P* = 0.0049) or plastid enzyme complexes (PTEC) (*χ*^2^ = 5.58, *P* = 0.018). Although the levels of retention within each category varied among species, we did not find a difference in the retention levels between the mitochondria-targeted versus plastid-targeted genes in any of the six species (*P* > 0.05 for all species). Wheat (*χ*^2^ = 18.35, *P* < 0.0001) and cotton (*χ*^2^ = 11.05, *P* = 0.00089) both exhibited significantly more PPR genes (relative to non-organelle-targeted genes) compared with the combined diploids, while the tobacco genome encoded significantly fewer PPR genes than expected (relative to non-organelle-targeted genes) compared with the combined diploids (*χ*^2^ = 68.09, *P* < 0.0001). Together, these results provide mixed support for the rapid loss of organelle-targeted genes compared with the rest of the genome in allopolyploids, but do indicate that similar forces may equally affect the mitochondria- and plastid-targeted genes.

**Fig. 3.**
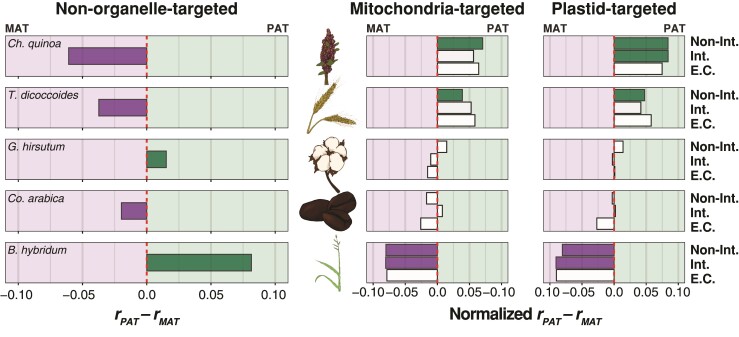
Gene content bias across allotetraploid subgenomes. The proportion of genes present in the paternal (*r_PAT_*) versus maternal (*r_MAT_*) subgenomes is depicted for each of five allotetraploid species arranged vertically from the oldest (top) to youngest (bottom). Tobacco was excluded from this analysis because the massive rearrangement it has experienced makes subgenomic identification based on chromosomal position intractable. The left panel includes only the non-organelle-targeted genes, the middle panel includes only the mitochondria-targeted genes, and the right panel includes only the plastid-targeted genes. In the left panel, the red-dashed line represents equal content across the subgenomes. In the right two panels, the *r_PAT_* and *r_MAT_* are normalized by the overall genome-wide gene number changes, excluding those genes targeted to organelles. Proportion deltas that depart significantly from the red line are filled in solid according to the direction of subgenomic bias (i.e., green: *r_PAT_* > *r_MAT_*; purple: *r_PAT_* < *r_MAT_*; no fill: *r_PAT_* ≈ *r_MAT_*). The intimacy of interactions is depicted on the *y*-axis for each of the right two panels from low or no interaction with organelle gene products (top), to interacting genes (middle), to genes involved in mitochondrial or plastid enzyme complexes (bottom).

A second possible consequence of polyploidy is the incompatibility between the paternally derived nuclear subgenome and the maternally derived cytoplasmic genomes, potentially resulting in the preferential loss of paternally derived organelle-targeted genes in hybrid (allo)polyploid species. This effect could exaggerate a general subgenome bias for paternal loss or partially compensate for a general bias toward maternal loss. For five of the allotetraploid genomes, it was possible to assign genes to subgenomes based on their chromosome of origin (i.e., not based on gene trees), thereby permitting a relative assessment of parental gene loss. The sole exception, *N. tabacum*, has experienced extensive genomic rearrangement between the subgenomes (e.g., chromosomal fusions, translocations) since polyploidization (Lim et al. 2004) that precludes subgenome assignment is based on the physical location. In general, we found significant differences in non-organelle-targeted gene abundance across the subgenomes for all five allotetraploid species (table 4), with quinoa, wheat, and coffee exhibiting more paternal homoeolog loss, whereas cotton and *Brachypodium* exhibit a deficit in maternal homoeologs (fig. 3, left panel). Interestingly, however, when considering biases in the organelle-targeted genes after correcting for genome-wide levels, these biases flip for quinoa, wheat, and *Brachypodium*. That is, while both quinoa and wheat exhibit a biased loss of *paternal* homoeologs for the non-organellar targeting genes, those that are targeted to the organelles exhibit biased *maternal* loss (again, relative to background; fig. 3 right panels, supplementary table S3, Supplementary Material online). Similarly, *Brachypodium* organelle-targeted genes exhibit biased paternal loss (relative to background), whereas the genome-wide pattern shows a biased maternal loss. These patterns were also found using the diploid relatives to correct for different gene abundances at the time of allopolyploidization (supplementary fig. S4, Supplementary Material online). While the maternal homoeolog deficit for the organelle-targeted genes found in wheat and quinoa is contrary to predictions based on cytonuclear incompatibilities, we note that this reflects homoeolog retention relative to the genome-wide rate and suggests that these species exhibit a lower degree of subgenomic bias in their organelle-targeted genes than the genome-wide rate.

**Table 4.** Biased Gene Content of the Non-organelle-targeted Genes Across the Subgenomes of Five Allotetraploid Angiosperms.

Species^a^	Diploid Paternal Model	Paternal Subgenome	Maternal Subgenome	Diploid Maternal Model	*r* *_PAT_* − *r_MAT_* (95% CI)^b,c^	Binomial test *p-*value
Quinoa	19,525	9,786	11,053	19,336	−0.061 (−0.074 to −0.047)	<0.0001
Wheat	32,734	48,786	52,571	46,164	−0.037 (−0.044 to −0.031)	<0.0001
Cotton	34,004	29,762	28,871	34,201	0.015 (0.007–0.023)	0.00024
Coffee	22,897	19,008	19,773	26,017	−0.020 (−0.030 to −0.010)	0.00011
*Brachypodium*	31,446	34,860	29,605	27,039	0.082 (0.074–0.089)	<0.0001

a Tobacco was excluded from this analysis because its subgenomes cannot be easily disentangled based on the chromosome number.

b *r* *_PAT_* refers to the ratio of genes found in the paternal subgenome relative to the paternal diploid model, and *r_MAT_* refers to the ratio of genes found in the maternal subgenome relative to the maternal diploid model.

c 95% CIs were inferred from the Exact binomial test in the R stats package, following Clopper and Pearson (1934).

### Evolutionary Rate Differences Across Subgenomes and Gene Functional Categories

We used the CyMIRA gene classifications from the maternal diploid models of each allotetraploid to classify single-copy orthologous quintets into functional gene categories, except in the case of wheat. For wheat, the paternal diploid model, *T. urartu*, was used because the maternal diploid model (i.e., *A. speltoides*) is only represented by a transcriptome. These functional categories served as the basis for our concatenated and gene-level analyses of evolutionary rate. Summary statistics describing the number of orthologous quintets in each functional category are presented for each allopolyploid system in table 5 and supplementary fig. S5, Supplementary Material online, along with the rates of synonymous (*d_S_*) and nonsynonymous (*d_N_*) evolution in concatenated alignments.

**Table 5.** Single-copy Orthologous Quintets Partitioned by Functional Category in Six Allotetraploid Species.

Species	Functional Category	Number of Quintets	*d_S_* ^a^	*d_N_* ^b^	*ω* ^c^	*ω* *_PAT_* (95% CI)^d^	*ω* *_MAT_* (95% CI)^e^
Quinoa	Non-organelle-targeted	6,885	0.499	0.096	0.193	0.290 (0.28–0.30)	0.332 (0.32–0.34)
	Mitochondria-targeted noninteracting	615	0.444	0.079	0.179	0.278 (0.25–0.31)	0.319 (0.29–0.35)
	Mitochondria-targeted interacting^f^	213	0.477	0.105	0.220	0.352 (0.29–0.42)	0.369 (0.31–0.43)
	Mitochondria enzyme complexes	69	0.465	0.084	0.180	0.279 (0.19–0.40)	0.350 (0.26–0.45)
	Plastid-targeted noninteracting	900	0.449	0.081	0.180	0.279 (0.25–0.30)	0.322 (0.30–0.35)
	Plastid-targeted interacting	212	0.463	0.091	0.197	0.290 (0.24–0.34)	0.356 (0.29–0.43)
	Plastid enzyme complexes	74	0.483	0.081	0.168	0.305 (0.15–0.40)	0.361 (0.21–0.47)
Wheat	Non-organelle-targeted	3,507	0.1882	0.035	0.187	0.444 (0.41–0.48)	0.201 (0.19–0.21)
	Mitochondria-targeted noninteracting	476	0.179	0.030	0.169	0.466 (0.38–0.55)	0.241 (0.20–0.28)
	Mitochondria-targeted interacting	67	0.162	0.033	0.206	0.651 (0.30–0.82)	0.215 (0.12–0.31)
	Mitochondria enzyme complexes^g^	38	0.191	0.039	0.206	0.661 (0.27–0.81)	0.077 (0.05–0.13)
	Plastid-targeted noninteracting	561	0.179	0.031	0.171	0.469 (0.39–0.54)	0.237 (0.20–0.28)
	Plastid-targeted interacting	86	0.171	0.030	0.175	0.353 (0.19–0.49)	0.278 ( 0.17–0.36)
	Plastid enzyme complexes	38	0.228	0.030	0.131	0.310 (0.09–0.53)	0.159 (0.06–0.27)
Cotton	Non-organelle-targeted	14,957	0.108	0.038	0.348	0.422 (0.41–0.43)	0.535 (0.52–0.55)
	Mitochondria-targeted noninteracting	1,076	0.106	0.033	0.309	0.391 (0.35–0.43)	0.480 (0.44–0.52)
	Mitochondria-targeted interacting	375	0.103	0.034	0.332	0.392 (0.35–0.44)	0.564 (0.48–0.65)
	Mitochondria enzyme complexes	100	0.119	0.037	0.310	0.376 (0.28–0.53)	0.568 (0.42–0.72)
	Plastid-targeted noninteracting	1,502	0.106	0.033	0.309	0.392 (0.36–0.43)	0.514 (0.48–0.56)
	Plastid-targeted interacting	270	0.102	0.031	0.303	0.352 (0.31–0.40)	0.525 (0.45–0.60)
	Plastid enzyme complexes	94	0.100	0.029	0.289	0.322 (0.23–0.46)	0.516 (0.38–0.68)
Coffee	Non-organelle-targeted	3,397	0.181	0.055	0.306	0.486 (0.45–0.52)	0.429 (0.40–0.46)
	Mitochondria-targeted noninteracting	306	0.181	0.051	0.281	0.533 (0.44–0.61)	0.548 (0.40–0.66)
	Mitochondria-targeted interacting	121	0.170	0.052	0.306	0.513 (0.39–0.65)	0.426 (0.33–0.55)
	Mitochondria enzyme complexes	31	0.187	0.057	0.307	0.597 (0.25–0.92)	0.482 (0.39–0.96)
	Plastid-targeted noninteracting	420	0.180	0.051	0.285	0.510 (0.42–0.57)	0.514 (0.39–0.61)
	Plastid-targeted interacting	88	0.163	0.049	0.300	0.438 (0.29–0.66)	0.537 (0.36–0.76)
	Plastid enzyme complexes	25	0.159	0.043	0.273	1.182 (0.23–5.17)	0.363 (0.08–1.44)
Tobacco	Non-organelle-targeted	7,323	0.438	0.090	0.205	0.522 (0.38–0.54)	0.631 (0.40–0.65)
	Mitochondria-targeted noninteracting	675	0.375	0.071	0.190	0.466 (0.40–0.53)	0.654 (0.58–0.73)
	Mitochondria-targeted interacting	209	0.374	0.082	0.220	0.490 (0.42–0.57)	0.628 (0.53–0.74)
	Mitochondria enzyme complexes	59	0.392	0.070	0.178	0.490 (0.37–0.61)	0.770 (0.50–1.37)
	Plastid-targeted noninteracting	952	0.380	0.072	0.191	0.470 (0.42–0.52)	0.628 (0.57–0.69)
	Plastid-targeted interacting	183	0.370	0.074	0.200	0.541 (0.41–0.71)	0.591 (0.47–0.73)
	Plastid enzyme complexes	72	0.406	0.070	0.173	0.736 (0.35–1.21)	0.604 (0.37–0.99)
*Brachypodium*	Non-organelle-targeted	11,886	0.449	0.105	0.234	0.328 (0.31–0.45)	0.347 (0.33–0.47)
	Mitochondria-targeted noninteracting	1,310	0.388	0.0759	0.196	0.318 (0.30–0.47)	0.327 (0.27–0.40)
	Mitochondria-targeted interacting	367	0.398	0.086	0.216	0.386 (0.27–0.38)	0.405 (0.30–0.49)
	Mitochondria enzyme complexes	116	0.399	0.0645	0.162	0.222 (0.14–0.34)	0.126 (0.36–0.24)
	Plastid-targeted noninteracting	1,497	0.389	0.0763	0.196	0.305 (0.26–0.37)	0.312 (0.26–0.38)
	Plastid-targeted interacting	256	0.396	0.0829	0.209	0.385 (0.24–0.49)	0.284 (0.20–0.39)
	Plastid enzyme complexes	83	0.485	0.0626	0.129	0.123 (0.06–0.22)	0.276 (0.08–0.50)

a *d_S_* here reflects the ML estimates of the total synonymous branch length of the entire tree from 1,000 gene-level bootstrap replicates (5 replicate runs per bootstrap replicate).

b *d_N_* here reflects the ML estimates of the total nonsynonymous branch length of the entire tree from 1,000 gene-level bootstrap replicates (5 replicate runs per bootstrap replicate).

c *ω* here reflects the ML estimates of quintet-wide *d_N_* relative to quintet-wide *d_S_*.

d *ω* *_PAT_* refers to the ML estimate of the *ω* value for the paternal subgenome branch, with 95% CIs obtained from 1,000 gene-level bootstrap replicates (5 replicate runs per bootstrap replicate).

e *ω* *_MAT_* refers to the ML estimate of the *ω* value for the maternal subgenome branch, with 95% CIs obtained from 1,000 gene-level bootstrap replicates (5 replicate runs per bootstrap replicate).

f Interacting genes are defined as those nuclear-encoded genes whose products interact with the mitochondrial/plastid genomes or gene products according to the CyMIRA classifications scheme (Forsythe et al. 2019).

g Significant result is likely due to poor alignment. See supplementary fig. S4, Supplementary Material online for more details.

The rates of protein-sequence evolution vary substantially across the CyMIRA functional categories, likely indicative of variation in functional constraint (supplementary fig. S5*a*, Supplementary Material online). In particular, protein sequences of the mitochondrial OXPHOS complexes, several of the plastid photosynthesis complexes (but not all, see e.g., the NADH dehydrogenase-like [NDH] complex), as well as the mitochondrial and plastid RNA polymerases appear to evolve especially slowly, indicating that they have experienced relatively stringent negative selection in these angiosperms. In addition to complex-level effects, we also observed differences in protein-sequence evolution across our focal angiosperm systems, with coffee and cotton genomes exhibiting higher quintet-wide *d_N_/d_S_* values compared with quinoa, wheat, tobacco, and *Brachypodium* (supplementary fig. S5*b*, Supplementary Material online).

**Fig. 4.**
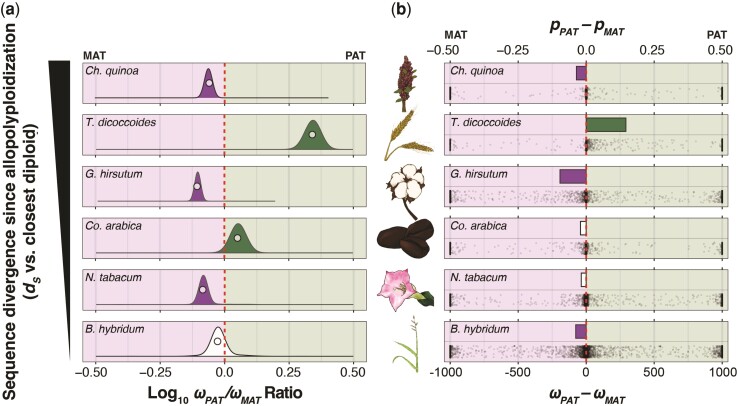
Genome-wide bias in *ω* (*d_N_/d_S_*) across the maternal and paternal subgenomes. (*a*) Log-transformed ratios of *ω* values in the paternal (*ω_PAT_*) versus maternal (*ω_MAT_*) subgenomes from concatenations (circles), and the underlying bootstrap distributions (density curves) of genes encoding proteins that are not targeted to either the plastids or mitochondria. Species panels are arranged vertically from the oldest (top) to the youngest (bottom). The red-dashed line indicates equal *ω* values across the subgenomes, the left side of each plot indicates higher *ω* values in the maternal subgenomes, and the right side of each plot indicates higher *ω* values in the paternal subgenome. The bootstrap distributions of *ω* ratios that depart significantly (*P* < 0.05) from the red line are filled in solid according to the direction of subgenomic bias (i.e., green: *ω_PAT_*/*ω_MAT_* > 1.0; purple: *ω_PAT_*/*ω_MAT_* < 1.0; no fill: *ω_PAT_*/*ω_MAT_* ≈ 1.0). (*b*) Estimates of *ω_PAT_* − *ω_MAT_* for each individual gene is depicted on the bottom half of each species’ panel and the proportion of genes with higher *ω* values in the paternal subgenome (*p_PAT_*) minus the proportion of genes with higher *ω* values in the maternal subgenome (*p_MAT_*) is depicted on the top half of each species’ panel for all genes not targeted to either the mitochondria or the plastids. The red-dashed line represents equal proportions of genes with higher *ω* values across subgenomes, and bars are filled in when proportion deltas are significantly different from zero (i.e., green: *p_PAT_* > *p_MAT_*; purple: *p_PAT_* < *p_MAT_*; no fill: *p_PAT_* ≈ *p_MAT_*).

Cytonuclear incompatibilities between the maternally derived cytoplasmic genomes and paternal subgenomes of allopolyploids are expected to result in accelerated rates of protein-sequence evolution in the paternal homoeologs of the organelle-targeted genes. We first tested for signatures of these cytonuclear incompatibilities by estimating differences in the rates of protein-sequence evolution (i.e., *d_N_/d_S_* = *ω*) in the concatenated and individual gene alignments of the paternal (*ω_PAT_*) versus maternal (*ω_MAT_*) subgenomes in the non-organelle-targeted (NOT) genes to assess whether genome-wide biases exist in our six focal allopolyploids. In the concatenated analyses, quinoa, wheat, cotton, and tobacco all showed significant departures (i.e., <2.5% overlap of bootstrap distributions between *ω_PAT_* and *ω_MAT_*) from equal rates of evolution across the subgenomes. In particular, quinoa, cotton, and tobacco exhibited higher *ω* values in the maternally derived homoeologs of the NOT genes than the paternal homoeologs (i.e., *ω_PAT_* : *ω_MAT_* ratio <1), while coffee and wheat showed the opposite pattern in which the paternally derived homoeologs exhibit faster rates of protein-sequence evolution than the maternal homoeologs (i.e., *ω_PAT_* : *ω_MAT_* ratio >1; fig. 4*a*). We observed similar patterns in the gene-level analyses as compared with the concatenated analyses in the three older polyploids (fig. 4*b*): a significantly higher proportion of the maternal homoeologs (*p_MAT_*) exhibited faster rates of evolution than the paternal homoeologs (*p_PAT_*) in quinoa (binomial test, *P* = 0.0022) and cotton (binomial test, *P* < 0.0001), while *p_PAT_* was significantly greater than *p_MAT_* in wheat (binomial test, *P* < 0.0001). Although *p_MAT_* was greater than *p_PAT_* in the concatenated analysis of tobacco subgenomes, the difference was not significant at the gene level (binomial test, *P* = 0.183). A similar result was obtained in coffee, with the concatenated analysis showing a significant paternal bias, but gene-level patterns did not appear to be paternally biased (binomial test, *P* = 0.375). The bootstrap distributions of *ω_MAT_* in *Brachypodium* estimated from concatenated alignments were higher than the bootstrap distributions of *ω_PAT_*, but were not significantly different (i.e., >2.5% overlap), while *p_MAT_* was significantly greater than *p_PAT_* at the individual gene level (binomial test, *P* = 0.00026). The higher *ω* values in the maternal subgenomes of quinoa, cotton, and *Brachypodium* and the higher *ω* values in the paternal subgenome of coffee were primarily driven by differences in *d_N_* as opposed to *d_S_* (fig. 2), indicating that these subgenomes experience different rates of protein-sequence evolution. By contrast, the elevated *ω* values in the maternal subgenome of tobacco and the paternal subgenome of wheat were primarily driven by *d_S_* (fig. 2), potentially indicating that different subgenomes experience different mutation rates or that the diploids used here represent highly asymmetric models of the diploid progenitors. Taken together, these analyses of the NOT genes indicate that allopolyploids experience significant biases in evolution rates across the subgenomes present inside the same cell.

**Fig. 5.**
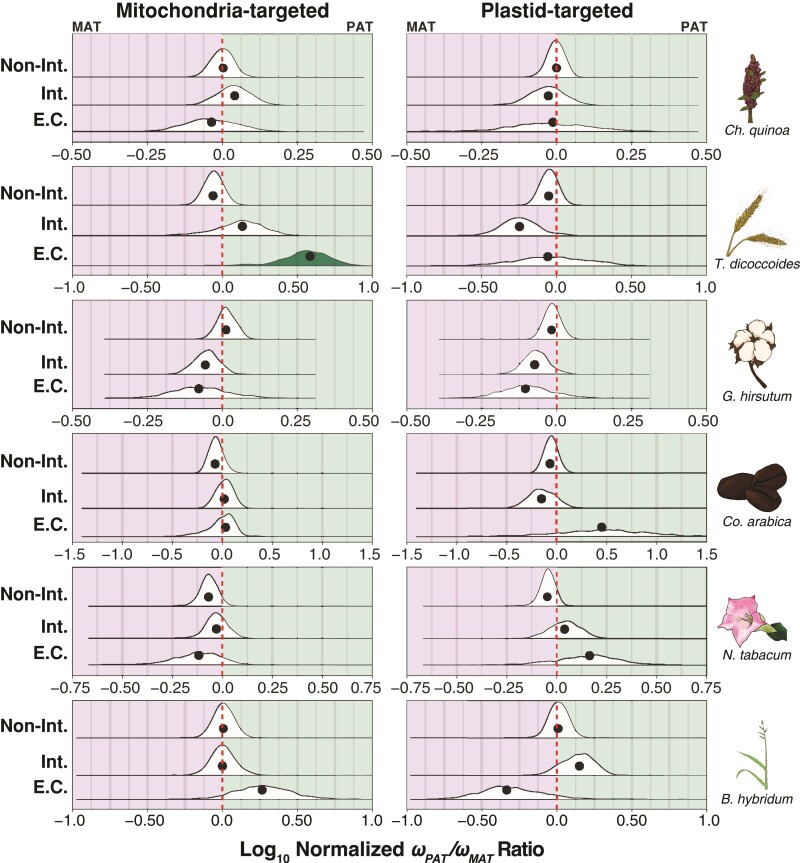
Ratios of maternal versus paternal subgenome *ω* values in the organelle-targeted genes. Log-transformed ratios of maternal versus paternal *ω* values from the terminal polyploid subgenome branches for concatenations (black circles) and underlying bootstrap distributions (density curves) of the mitochondria- (left) and plastid-targeted (right) genes in six focal allotetraploid species. Species panels are arranged vertically from the oldest (top) to the youngest (bottom). The red-dashed line indicates the *ω_PAT_*/*ω_MAT_* ratio for a concatenation of genes not targeted to the organelles (fig. 4*a*). Ratios left of the red line indicate higher *ω* values in the maternal subgenome, and ratios right of the red line indicate higher *ω* values in the paternal subgenome, after accounting for genome-wide patterns. Bootstrap distributions of *ω* ratios that depart significantly (*P* < 0.05) from the red line are filled in solid according to the direction of subgenomic bias (i.e., green: normalized *ω_PAT_*/*ω_MAT_* > 1.0; purple: normalized *ω_PAT_*/*ω_MAT_* < 1.0; no fill: normalized *ω_PAT_*/*ω_MAT_* ≈ 1.0). The intimacy of interactions is indicated on the *y*-axis from low or no interaction with organelle gene products (top), to interacting genes (middle), to genes involved in mitochondrial or plastid enzyme complexes (bottom).

We next performed the concatenated and gene-level analyses of *ω_PAT_* and *ω_MAT_* in organelle-targeted genes (normalized by the NOT genes) to test whether the paternal homoeologs exhibited faster rates of protein-sequence evolution than the maternal homoeologs, as predicted if the paternal subgenomes harbor incompatibilities with the cytoplasmic genomes. We found evidence that the concatenations of wheat genes involved in MTECs exhibited significantly higher *ω_PAT_* values (median = 0.661, 95% CI = 0.268–0.807) compared with *ω_MAT_* values (median = 0.0771, 95% CI = 0.0460–0.125), relative to the NOT genes (*ω_PAT_* = 0.444, 95% CI = 0.414–0.476; *ω_MAT_* = 0.201, 95% CI = 0.189–0.215); however, no other species or functional classes exhibited the predicted pattern (fig. 5). To further investigate the patterns of molecular evolution in the wheat MTEC genes, we manually inspected and trimmed concatenated alignments from the NOT genes, MTEC genes, and PTEC genes and re-inferred *ω_PAT_* and *ω_MAT_* in all three gene categories. Importantly, we found two small regions from two genes in the MTECs that were poorly aligned only in the paternal subgenome, contributing to elevated *ω_PAT_* but not *ω_MAT_*. The poorly aligned regions appeared to be caused by a combination of an apparent frameshift in the paternal homoeologs of one gene encoding a protein involved in the NADH dehydrogenase (OXPHOS Complex I—TRIDC1AG048530) and another gene encoding a protein that functions in a large subunit of the mitoribosome (TRIDC4AG029590) had an exon on the 3′ end of the gene with no apparent homology to the other sequences in the quintet (likely due to misannotation or misassembly, as the new *T. turgidum* assembly, GCA_900231445.1, does not have this same issue). Both genes exhibited substantially different *d_S_* and *d_N_* values compared with other genes in the same functional gene category (supplementary table S4, Supplementary Material online). Trimming the poorly aligned regions resulted in a substantially lower *d_N_* value for the concatenated alignments of MTEC genes, which in turn, caused a lower *ω_PAT_* value that was not significantly different from the *ω_MAT_* value (supplementary fig. S6, Supplementary Material online). All trimmed alignments and analyses are available at https://github.com/jsharbrough/allopolyploidCytonuclearEvolutionaryRate. For the gene-level analyses, we did not find any functional categories in any species that exhibited significantly different normalized proportions of genes with higher *ω_PAT_* or *ω_MAT_* (supplementary fig. S7, Supplementary Material online), a pattern that did not change when *d_N_* was used in the place of *ω*. Thus, global accelerations do not appear in the protein-sequence evolutionary rate of the paternal homoeologs of organelle-targeted genes in the wake of allopolyploidization.

We next evaluated *ω* values at the level of specific cytonuclear interactions (supplementary table S5, Supplementary Material online) and found scattered patterns of both paternal and maternal bias across various cytonuclear interactions in the three older polyploids (i.e., quinoa, wheat, and cotton). In particular, the paternal homoeologs of quinoa exhibited significantly higher *ω* values (i.e., *ω* values from concatenated alignments ±1 SE were outside bootstrap-constructed 95% CIs of the NOT genes) than the maternal homoeologs in mitochondrial tRNA base modification, plastid NDH, and plastid tRNA base modification, and the maternal homoeologs exhibited significantly higher *ω* values than the paternal homoeologs in both the subunits of chlororibosome and Photosystem I (PSI). As seen at higher levels of the organization, wheat MTECs generally exhibited higher *ω* values in the paternal versus maternal homoeologs (see below for detailed discussion) compared with the NOT genes. However, the reverse was true in PTEC genes, with plastid PSII exhibiting significantly higher *ω* values in the maternal versus paternal homoeologs, relative to the NOT genes. Wheat organellar tRNA aminoacyl synthetases, which are largely dual-targeted (Duchêne et al. 2005), also exhibited a significant maternal bias compared with the NOT genes. Cotton had fewer CyMIRA categories that showed evidence of bias over-and-above genome-wide levels, with just the mitochondria- and plastid-targeted recombination, replication, and repair genes (also commonly dual-targeted; Forsythe et al. 2019) exhibiting elevated *ω* values in the paternal versus maternal homoeologs and also the large subunit of the mitoribosome and mitochondria-targeted PPR genes exhibiting higher *ω* values in them compared to the NOT genes. Coffee, tobacco, and *Brachypodium* all appear to be too young for this analysis, as only a single functional category (plastid transcription and transcript maturation) in coffee showed significant (maternal) bias compared with the NOT genes, despite genome-wide bias in the *ω* values of coffee and tobacco. There were no CyMIRA categories that exhibited consistent patterns across even the older three allopolyploids, highlighting the highly context-specific nature of evolutionary dynamics of cytonuclear interactions in allopolyploids.

Because incompatibilities are only likely to arise in genes that are divergent at the time of allopolyploidization, we also performed the analyses described above on high and low-divergence gene bins. To do so, we split single-copy orthologous quintets into two groups: those with high amino acid sequence divergence between the diploid models (measured by *d_N_*) and those with low amino acid sequence divergence. We used a similar approach as before to normalize *ω_PAT_* and *ω_MAT_* using the NOT genes. There were only two cases in which high and low-divergence classes differed by more than one standard error: mitochondrial and PTEC of wheat (supplementary fig. S8, Supplementary Material online). In particular, the low-divergence class of wheat MTECs exhibited more extreme paternal bias than the high-divergence class, while the low-divergence class of wheat PTEC exhibited a more extreme maternal bias compared with the high-divergence class. This somewhat surprising result, notwithstanding the lack of signal in the high-divergence classes across the other functional categories and species, indicates that the cytonuclear incompatibilities of allopolyploids are not resolved by the faster rates of protein-sequence evolution in the paternal homoeologs.

We compared the patterns of autapomorphic amino acid changing mutations at sites that were conserved throughout the rest of the quintet in genes encoding the subunits of mitochondrial enzyme complexes. For each species, we observed several gene functional categories that exhibited an excess number of autapomorphic amino acid changes compared with genes not targeted to the mitochondria or plastids in one subgenome compared with the other. However, the direction of excess was not consistent across species or even across functional gene categories (supplementary table S6, Supplementary Material online).

Because the derived amino acids with substantially different biochemical properties compared with ancestral residues (i.e., radical amino acid changes) are especially likely to alter the protein structure and function (Perutz et al. 1965; Grantham 1974; Lesk and Chothia 1980; Nakashima et al. 1986; Rumbley et al. 2001; Boyko et al. 2008), we next restricted these autapomorphy analyses of derived amino acid changes in the tetraploids to include radical amino acid changes only (as defined by the conservative/radical index [CRI]; Sharbrough et al. 2018). As was the case with total derived amino acid changes, there existed several functional categories in each species that exhibited significant biases in the accumulation of radical autapomorphies across subgenomes, but the direction of bias and the functional categories identified were not consistent across species. Several notable functional categories did exhibit bias across multiple species though (e.g., DNA replication, recombination, and repair genes [quinoa, cotton, *Brachypodium*], tRNA base modification genes [quinoa, cotton, coffee, *Brachypodium*], and tRNA aminoacyl synthetases [wheat, tobacco]), potentially indicating they are hotbeds for cytonuclear incompatibilities and/or diploidization. Together, these results indicate that the cytonuclear enzymes exhibit complex- and species-specific patterns of accumulation of the derived amino acids at conserved sites.

In sum, our concatenated, gene-level, and site-level analyses provide evidence that the protein sequences of different allopolyploid subgenomes exhibit different *ω* values, potentially as a result of the different rates of protein-sequence evolution, but cytonuclear incompatibilities resulting from the allopolyploidization event do not leave global signatures of accelerated protein-sequence evolution in the paternal homoeologs of organelle-targeted genes. Moreover, while the organelle-targeted genes are often lost at higher rates than the genome-wide rates of diploidization, this is not always the case, especially in cotton, and the biased gene content of allopolyploid subgenomes does not appear to be related to cytonuclear incompatibilities. Rather, only species- and complex-specific cytonuclear dynamics appear to alter the rates of evolution in organelle-targeted genes, and in directions not uniformly consistent with allopolyploidy induced cytonuclear incompatibilities.

## Discussion

We inferred orthologous gene sets, partitioned genes by subcellular targeting localization and cytonuclear interaction, and evaluated the genome-wide patterns of gene content and natural selection across the subgenomes of six allotetraploid angiosperms. We report significant genome-wide biases across the maternal versus paternal subgenomes in the overall gene content in all five allopolyploids tested and in the mutation-rate-corrected rates of protein-sequence evolution (i.e., *ω*) in all six allopolyploid genomes tested. The directions of bias in both the gene content and higher *ω* were not consistent across independent allopolyploidization events, and the patterns observed in gene content did not appear to be similar in direction as biased in *ω*.

The analyses reported here support three primary conclusions: (1) allopolyploid subgenomes exhibit substantially different rates of protein-sequence evolution from one another despite existing inside the same cell for thousands to millions of years; (2) cytonuclear incompatibilities between the cytoplasmic genomes and the paternal subgenome are complex and taxon-specific and do not result in global increases in the rates of protein-sequence evolution in paternal homoeologs of the organelle-targeted genes; and (3) gene content is not equally distributed across subgenomes, with both species and cytonuclear functional classes contributing to variation in the rate at which genomes fractionate following WGDs. The foregoing conclusions suggest many questions that have implications for our understanding of polyploid biology.

### Differential Rates of Protein-Sequence Evolution Across Allopolyploid Subgenomes

Most prominent among our data are the remarkable differences in evolutionary patterns across the subgenomes, raising the question of what evolutionary forces underlie these subgenomic biases? That is, allopolyploid subgenomes that have been (co-)evolving inside the same nucleus for thousands to millions of years (Gaeta and Pires 2010) remain on separate evolutionary trajectories with respect to the evolutionary rates in protein-coding genes. Here, we consider several phenomena that could play a role in establishing and maintaining subgenomic biases.

If *ω* is adequately inferring the patterns of natural selection across subgenomes (but see below for alternative explanations), then the patterns of subgenomic biases in the rates of protein-sequence evolution reported here could arise from differences in the efficacy of selection or effective population size (*N_e_*) across the subgenomes. In particular, genes that are more highly expressed (Drummond et al. 2005; Yang et al. 2012), have higher local recombination rates (Hill and Robertson 1966; Felsenstein 1974; Liu et al. 2017; Zhou et al. 2017), or lower local TE densities (Hollister and Gaut 2009; Freeling et al. 2012; Bird et al. 2018) (but see Wyler et al. 2020) are expected to experience increased efficacy of natural selection, and thus, exhibit reduced rates of protein-sequence evolution (Charlesworth 2009). That is, genome-wide differences between the progenitors at the time of allopolyploid formation (e.g., transcriptome size, recombination rate, TE load) would not only be expected to give rise to subgenomic differences in the immediate aftermath of polyploidization (Song et al. 1995, 2020; Koh et al. 2010; Parisod et al. 2010; Szadkowski et al. 2010), but could contribute to evolved differences across the subgenomes (Adams et al. 2003; Mutti et al. 2017; Cheng et al. 2018; Emery et al. 2018; Wendel et al. 2018; Wicker et al. 2018; Liu et al. 2020).

Mutation rate varies tremendously across species, populations, individuals, and even within genomes (Drake et al. 1998; Baer et al. 2007; Lynch 2010; Weng et al. 2019), making it a potential candidate for generating subgenome biases in *ω* (Kryazhimskiy and Plotkin 2008) if elevated mutation rate results in increased rates of background selection, thereby reducing *N_e_* (Charlesworth 2009). Such mutational biases across the subgenomes could reflect ancestral differences in parental species (e.g., differences in DNA methylation; Edger et al. 2017; Weng et al. 2019; Alger and Edger 2020), or could potentially arise after polyploidization in association with other biased phenomena such as recombination (Pelé et al. 2018), gene expression (Chen 2007; Akhunova et al. 2010; Flagel and Wendel 2010; Grover et al. 2012a; Yoo et al. 2013; Li et al. 2014; Liu et al. 2014; Hu et al. 2016; Wang et al. 2016; Edger et al. 2017; Nomaguchi et al. 2018), epigenetic marks (Madlung et al. 2002; Salmon et al. 2005; Shcherban et al. 2008; Fulneček et al. 2009; Akagi et al. 2016; Chen et al. 2017a; Song et al. 2017; Ding and Chen 2018), or transposable element activity (Senerchia et al. 2016; Springer et al. 2016; Vicient and Casacuberta 2017; Bird et al. 2018; Nieto Feliner et al. 2020), which are all thought to be mutagenic (Gonzalgo and Jones 1997; Bennetzen and Wang 2014; Jinks-Robertson and Bhagwat 2014; Halldorsson et al. 2019).

Subgenomes might also differ in *N_e_* as a result of backcrossing, in which one polyploid subgenome experiences higher rates of introgression than the other (Slotte et al. 2008; Zohren et al. 2016; Denton et al. 2018). Repeated allopolyploid formation or gene flow from diploids (e.g., *Brachypodium hybridum*—Gordon et al. 2020, *Arabidopsis suecica*—Novikova et al. 2017) can cause *N_e_* to differ across subgenomes. Finally, recombination could also act to bias inferences of *ω* artifactually because genetic material is exchanged across subgenomes via homoeologous exchange (Xiong et al. 2011; Albertin and Marullo 2012; Cenci et al. 2012; Chester et al. 2012; Chalhoub et al. 2014; Guo et al. 2014; Allendorf et al. 2015; He et al. 2017; Jarvis et al. 2017; Chen et al. 2018; Lloyd et al. 2018; Bertioli et al. 2019; Edger et al. 2019; Li et al. 2019b; Mason and Wendel 2020; Wu et al. 2020; Zhang et al. 2020), gene conversion (Wendel et al. 1995; Kovarik et al. 2004, 2005; Gaeta and Pires 2010; Salmon et al. 2010; Page et al. 2013; Chalhoub et al. 2014; Gong et al. 2014; Guo et al. 2014; Leal-Bertioli et al. 2018; Li et al. 2020; Liu et al. 2020), and other recombinational mechanisms (e.g., Mandáková et al. 2019) would be expected to bias *ω* inferred across a topologically constrained tree. However, we took steps to prevent this type of artifact from influencing our data by only including genes that exhibited gene-tree topologies that were consistent with the species tree topology.

The relative contributions of these various evolutionary dynamics are of central importance to the understanding of polyploid genomes, but testing each hypothesis, in turn, is made difficult by the fact that the sampled diploids are, to varying degrees, imprecise models of the ancestral progenitors. Therefore, an unknown fraction of each terminal “polyploid” branch in our quintet trees actually represents evolution as a diploid prior to hybridization. Wheat, in particular, is susceptible to artifactual inflation of *ω* because *A. speltoides* is so much more distantly related to the B subgenome of the polyploid than *T. urartu* is to the A subgenome (fig. 2). The persistence of deleterious changes since the divergence of the A subgenome and the diploid A genome may result in the overestimation of *ω* in the A subgenome compared with the B subgenome. The same logic applies to all of our polyploid taxa to varying extents; however, it is worth noting that while differences in *d_S_* across subgenomes were the primary drivers of differences in *ω* in wheat and tobacco, *d_N_* had a proportionally larger effect than *d_S_* on differences in *ω* in quinoa, cotton, coffee, and *Brachypodium*. This latter finding is consistent with selection being the driving factor in the evolutionary rate variations across subgenomes (but see prior caveat regarding the quality of diploid models and evolution prior to polyploidization), rather than mutation rate variation or the artifactual inflation of *ω* in the more closely related diploid-subgenome pair (discussed below). In the same vein, coffee and cotton, which are both thought to have extremely small effective population sizes (Simone et al. 2020; Yuan et al. 2021), exhibited the highest overall *ω* values (supplementary fig. S5*b*, Supplementary Material online).

Although the time since allopolyploidization is equal across both the subgenomes, the time since the diploid models of each subgenome diverged from the true diploid progenitors is (1) unknowable from this dataset (and is a ubiquitous confounding factor in allopolyploid formation inferences), and (2) different across the two diploid models for each system. This is especially true for the older allopolyploids (i.e., cotton, wheat, and quinoa). Importantly, we expected that the subgenome with the more closely related diploid model (i.e., lower *d_S_*) would exhibit artificially inflated *d_N_* (and therefore *ω*) relative to the other subgenome due to the persistence of slightly deleterious changes, as well as the masking effect acting on recessive deleterious changes in the allopolyploids (Conover and Wendel 2021). This is indeed the case for wheat, cotton, and tobacco, in which the subgenome with the more closely related diploid progenitor (fig. 2) exhibits elevated *ω* (fig. 4), but does not hold for quinoa, coffee, or *Brachypodium*. From this we can surmise that asymmetry in the quality of the diploid models has some effect on our observations (e.g., wheat), but it does not explain the entirety of our observations of differential *ω* across subgenomes (e.g., quinoa, coffee). Moreover, while the asymmetry in diploid models would be expected to obscure our ability to observe a cytonuclear effect, as the genome-wide effects are larger, we see no evidence of cytonuclear impact on the global *ω* values, even in systems with relatively little uncertainty or asymmetry in the diploid models.

**Fig. 6.**
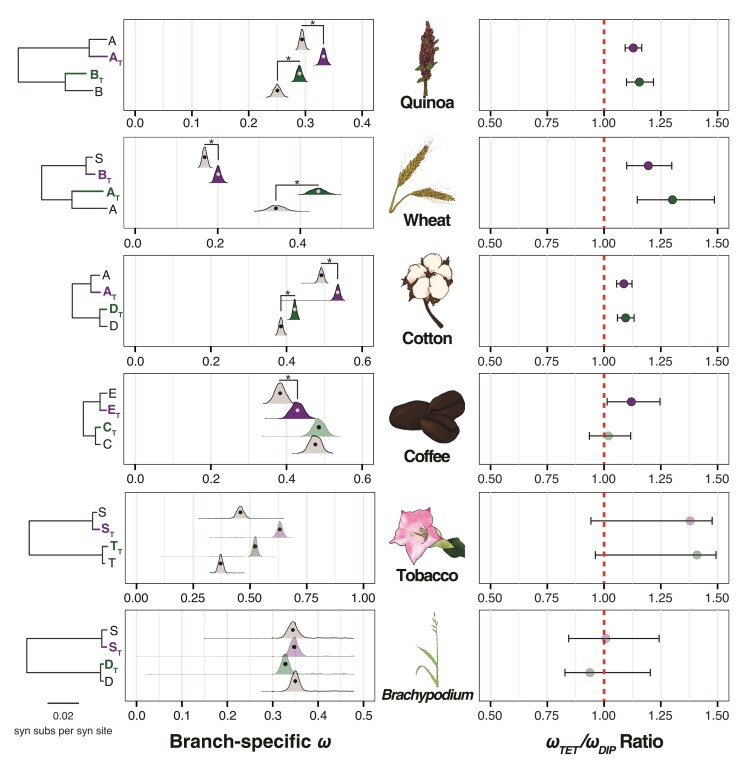
Comparison of branch-specific *ω* values in the polyploid subgenomes compared with their respective diploid models. Left: Maximum likelihood estimates (circles) and block-bootstrap distributions (density curves) of branch-specific *ω* values in the maternal (purple) and paternal (green) polyploid subgenomes compared with their diploid relatives (gray), with species arranged from the bottom to the top by increasing time since polyploidization. Trees to the left of the plots depict diploid polyploid relationships, with branch lengths representing synonymous substitution rates (i.e., *d_S_*), as shown in the left side of fig. 2. Statistical comparisons were made by calculating *ω* ratios for each bootstrap replicate and testing for overlap of 95% CIs with 1.0. Cases in which polyploid subgenomes significantly differ from their diploid relative are denoted by an asterisk and by a dark fill of the polyploid density curve. Right: Maximum likelihood estimates (circles) and 95% CIs inferred by the block-bootstrapping (error bars) of branch-specific *ω* ratios between the polyploid subgenomes and diploid relatives. The dashed red line represents equal *ω* values across comparisons.

To further explore relationships between the polyploid subgenomes and diploid models, we also compared branch-specific *ω* values in polyploid subgenomes to their respective diploid relatives. With the exceptions of *Brachypodium*, the youngest allopolyploid considered here, Nicotiana, and the paternally derived subgenome of polyploid coffee, polyploid subgenomes exhibit significantly higher *ω* values than their diploid relatives (fig. 6). This potentially indicates that the species of higher ploidy may inherently have higher *ω* due to mutations at phylogenetically conserved sites accumulating more rapidly in the polyploid subgenomes as a result of mutational masking (Conover and Wendel 2021). The extent to which the evolutionary trajectory of polyploid lineages is affected by the accelerated accumulation of deleterious mutations, therefore, represents an important open question in plant biology.

### No Global Signature of Mitonuclear Incompatibilities in the Paternal Homoeologs of Allopolyploid Genomes

To test the hypothesis that incompatibilities stemming from evolutionary mismatches between the maternally derived cytoplasmic genomes and the paternally derived nuclear subgenome result in preferential loss and accelerated rates of protein-sequence evolution in the paternal homoeologs of organelle-targeted genes, we applied the same analyses described above to the sets of CyMIRA-partitioned genes, after accounting for genome-wide effects. We did not discover evidence that cytonuclear incompatibilities shape either gene content or protein-sequence evolution in the paternal homoeologs of organelle-targeted genes, despite multiple distinct tests of this hypothesis. In particular, the patterns of gene content on the organelle-targeted genes exhibited an opposite pattern as that observed in the NOT genes in three of five allopolyploid taxa (the remaining two were not significantly different from genome-wide patterns), indicating that the organelle-targeted genes tend to exhibit greater balance across subgenomes than the rest of the genome. While the proportion of organelle-targeted genes per subgenome did not appear to be especially maternally biased, four of six allotetraploids had reduced overall proportions of the organelle-targeted genes compared with the NOT genes. Overall, the rates of protein-sequence evolution in the organelle-targeted and interacting genes generally reflected the genome-wide patterns of bias observed in the NOT genes, rather than rate accelerations peculiar to paternal but not the maternal homoeologs.

One outstanding question stemming from our analyses of protein-sequence evolution in the paternal versus maternal homoeologs of the organelle-targeted genes is why hybrid polyploid genomes appear to generally lack the genome-wide signatures of cytonuclear incompatibilities, despite their apparent importance in homoploid hybridization (Postel and Touzet 2020) and introgression events (Burton and Barreto 2012)? It is possible that cytonuclear incompatibilities do leave signatures on genomes, but not in terms of the accelerated rates of protein-sequence evolution in the paternal homoeologs. For example, pseudogenization may be a rapid and common mechanism for adaptation in plant genomes (e.g., Monroe et al. 2018), which would be missed by our quintet analyses. While we did not observe maternally biased gene content in the CyMIRA datasets, a direct analysis of bias in homoeologous pairs in which one copy is pseudogenized is necessary to rule out gene loss as a mechanism by which cytonuclear incompatibilities are resolved. The seemingly stochastic patterns of homoeolog bias in the accumulation of autapomorphic amino acid changes indicate that there are often cases in which the homoeologs of cytonuclear interacting genes evolve very differently, perhaps reflecting cytonuclear incompatibilities or the precursor to gene loss and diploidization, but these biases do not appear to coincide with the allopolyploidization events in any systematic way. The presence of biased accumulation of the autapomorphies in *Brachypodium* may indicate that cytonuclear incompatibilities are resolved rapidly. Cytonuclear incompatibilities may also be resolved via biased homoeolog expression (Grover et al. 2012a), gene conversion (Gong et al. 2014; Li et al. 2020), homoeologous exchange (Mason and Wendel 2020), subfunctionalization of subcellular localization by differential isoform usage across homoeologs (Qiu et al. 2020), or other potential mechanisms that would not generate global signatures of paternal acceleration in the coding sequences of organelle-targeted quintets.

Biased homoeolog expression represents a potential mechanism by which allopolyploids could resolve cytonuclear incompatibilities, but has found mixed support in the studies that have so far attempted it. In particular, cotton, tobacco, *Arabidopsis*, peanut, and the extremely young allotetraploid *Tragopogon miscellus* exhibit biased maternal expression of the nuclear-encoded subunit of Rubisco (Gong et al. 2012, 2014; Sehrish et al. 2015), but others have not found similar patterns in rice (Wang et al. 2017) or *Brassica napus* (Ferreira de Carvalho et al. 2019). Moving forward, large-scale genome-wide homoeolog expression bias could be evaluated across all the CyMIRA gene sets (not just Rubisco) to test this hypothesis. Additionally, the topological and alignment filtering steps we imposed on quintets here had the intended side effect of filtering out genes exhibiting gene conversion or homoeologous exchange. Notable among them was *rbcS*, which encodes the small subunit of Rubisco and was missing from filtered, single-copy quintets in five of six species complexes (present only in *Brachypodium*, the youngest allopolyploid). It is likely that because of *rbcS*’ propensity for gene conversion (Gong et al. 2014), this apparent “hotbed” for cytonuclear incompatibilities might provide additional evidence that was missed here. Certainly, a careful analysis of maternal versus paternal bias in gene conversion tracts and homoeologous exchanges among organelle-targeted genes may be a fruitful future approach.

An additional and perhaps likely possibility is that the cytoplasmic genomes of these allopolyploids may evolve too slowly in protein-coding sequence to generate widespread incompatibilities in hybrid polyploids (Wolfe et al. 1987). The relatively young allopolyploid *Brassica napus* may be a relevant example. The plastid genomes of *Brassica oleracea* and *Brassica rapa* have very few differences, and a recent analysis did not detect extensive incompatibilities with nuclear subgenomes (Ferreira de Carvalho et al. 2019). By contrast, the elevated rates of protein-sequence evolution and *ω* values in the organelle-interacting genes have been detected repeatedly in lineages with rapidly evolving cytoplasmic genomes (Osada and Akashi 2012; Barreto and Burton 2012; Sloan et al. 2014; Havird et al. 2015; Zhang et al. 2015; Rockenbach et al. 2016; Weng et al. 2016; Havird et al. 2017; Barreto et al. 2018; Yan et al. 2019; Forsythe et al. 2020). Therefore, the genome-wide analyses of evolutionary rates appear to be sensitive enough to detect cytonuclear incompatibilities when their effects are strong.

Because cytonuclear interactions are critical for hybrid lineage success in many cases (Burton et al. 2013; Bock et al. 2014; Dai et al. 2016), allopolyploids with cytonuclear incompatibilities may also be evolutionarily short-lived, such that the relatively successful allopolyploids assayed here may be unlikely to exhibit cytonuclear incompatibilities. Along these lines, allopolyploid unisexual salamanders do not appear to exhibit maternally biased expression of the nuclear-encoded OXPHOS genes (McElroy et al. 2017), despite the high rates of mitochondrial DNA sequence evolution and ancient divergence of the mitochondrial lineage from the paternal lineages (Denton et al. 2018). The high incidence of asexuality and selfing species among polyploid lineages may speak to this possibility (Otto and Whitton 2000). Overall, the data presented here and elsewhere appear most consistent with a scenario in which cytonuclear incompatibilities have minimal effects on the rates of protein-sequence evolution in allopolyploid plants.

### Cytonuclear Gene Content Evolution in Allopolyploids

Polyploids often have both larger cells (Butterfass 1987; Beaulieu et al. 2008; Marshall et al. 2012; Doyle and Coate 2019; Roddy et al. 2019) and more chloroplasts per cell in leaf tissue (Rhoades and Dempsey 1966; Bingham 1968; Krishnaswami and Andal 1978; Bowman 1986; Warner and Edwards 1993; Kawade et al. 2013; Bomblies 2020). Together, these phenomena suggest that stoichiometry between the nuclear and cytoplasmic genomes is important for cellular and organismal function (Sharbrough et al. 2017). Previous studies investigating single-copy genes in plants indicated that the organelle-targeted genes are among the first to return to diploidy following WGD events (De Smet et al. 2013; Li et al. 2016). By contrast, Ferreira de Carvalho et al. (2019) reported higher levels of maintained duplicates in organelle-targeted genes in the allopolyploid *Brassica napus*, compared with genome-wide levels. The gene content analyses presented here generally agree with this latter result, in that the organelle-targeted genes tend to be maintained in duplicated form than the rest of the genome in quinoa, wheat, and *Brachypodium*, although cotton and coffee offer important exceptions that muddy the waters. Notably, our analyses of gene content evolution did not explicitly identify the maternal or paternal homoeolog using gene trees, but instead, relied on physical position within the genome to assign ancestry. Still, we think it is unlikely that gene conversion or homoeologous exchange could adequately explain our observations, especially considering that the vast majority of single-copy genes feature gene-tree topologies consistent with the species tree in all taxa (supplementary table S7, Supplementary Material online). The discrepancies between the former two (performed in diploids) and the latter two (performed in polyploids) studies indicate that cytonuclear stoichiometry may be highly responsive to nuclear gene content. In support of that hypothesis, diverse polyploids appear to compensate for elevated nuclear ploidy with increased organelle genome copy number (Whiteway and Lee 1977; Dean and Leech 1982; Bowman 1986; Oberprieler et al. 2019; Coate et al. 2020; Gyorfy et al. 2021). Additional studies investigating the immediate and evolved consequences of cytonuclear stoichiometry at the genomic, transcriptomic, proteomic, and organellar levels, especially by homoeologous pair analysis, will provide valuable insights into the unresolved question of how genome doubling can affect cellular energy production and homeostasis.

### Summary

The genome-wide analyses of maternal versus paternal evolutionary rates presented here represent the most extensive investigation of cytonuclear incompatibilities in allopolyploids performed to date, representing six distinct allopolyploidization events of varying ages and divergences. We find clear evidence of differential evolution across the subgenomes, but little evidence of paternal homoeolog-specific accelerations of evolutionary rates in the organelle-targeted genes. Additionally, we found that the organelle-targeted gene content tends to be less biased than the rest of the genome, with mixed evidence of whether the organelle-targeted genes more likely tend to be lost more often than the rest of the genome. Further study investigating the forces underlying these observations and the consequences for organismal energy metabolism and homeostasis will be critical for understanding the cytonuclear dimension of allopolyploidy.

## Materials and Methods

### Genomic Datasets

The proliferation of genome assemblies for polyploid plants and their diploid relatives has enabled powerful phylogenomic analyses. We identified that six allotetraploids that share hybrid origins (fig. 1*a*) have publicly available chromosome-scale genome assemblies for both the polyploid and diploids that are most closely related to each subgenome (with the exception of the wild emmer wheat [*T. dicoccoides*] B subgenome, whose diploid relative [*A. speltoides*] only has a transcriptome available), and varying degrees of divergence between their diploid progenitors and the amount of time since allopolyploidization (fig. 1*b*). We also included the closest available chromosome-scale assembly for an outgroup species to polarize substitutions. Accession numbers and references are provided for assemblies and annotations used from each species complex in table 6.

**Table 6.** Genomic Resources for Six Allotetraploid Species Complexes.

Species complex	Species	Ploidy	Version/Accession	Reference
*Brachypodium*	*Hordeum vulgare* ^a^	2x	GCA_901482405.1	Mascher et al. (2017)
	*Brachypodium distachyon*	2x	GCA_000005505.4	Gordon et al. (2017)
	*Brachypodium stacei* ^b^	2x	B_stacei_v1_1	Gordon et al. (2020)
	*Brachypodium hybridum*	4x	B_hybridum_v1_1	Gordon et al. (2020)
Coffee	*Gardenia jasminoides* ^a^	2x	GCA_013103745.1	Xu et al. (2020)
	*Coffea canephora*	2x	GCA_900059795.1	Denoeud et al. (2014)
	*Coffea eugenioides* ^c^	2x	GCA_003713205.1	Gaitán et al. (2019)
	*Coffea arabica*	4x	GCA_003713225.1	Tran et al. (2018); Gaitán et al. (2019)
Cotton	*Gossypioides kirkii* ^a^	2x	Gossypioides_kirkii_ISU-v3.0	Udall et al. (2019b)
	*Gossypium raimondii*	2x	G.raimondii_JGI_221_v2.0	Paterson et al. (2012)
	*Gossypium arboreum* ^d^	2x	G.arboreum_CRI-A2_assembly_v1.0	Jia et al. (2018)
	*Gossypium hirsutum*	4x	Ghirsutum_458_v1.0	Saski et al. (2017)
Quinoa	*Spinacia oleracea* ^a^	2x	GCA_002007265.1	Xu et al. (2017)
	*Chenopodium suecicum*	2x	Csuecicum_DT_PBjellyM2	
	*Chenopodium pallidicaule* ^e^	2x	PGA_assembly_final_assembly_Cpallidicaule	Mangelson et al. (2019)
	*Chenopodium quinoa*	4x	quinoa_pb_chicago-2-final_PBJELLY_pilon	Jarvis et al. (2017)
Tobacco	*Solanum lycopersicum* ^a^	2x	ITAG4.0	Hosmani et al. (2019)
	*Nicotiana tomentosiformis*	2x	GCA_000390325.2	Sierro et al. (2013)
	*Nicotiana sylvestris* ^f^	2x	GCA_000393655.1	Sierro et al. (2013)
	*Nicotiana tabacum*	4x	GCA_002210045.1	Edwards et al. (2017)
Wheat	*Hordeum vulgare* ^a^	2x	GCA_901482405.1	Mascher et al. (2017)
	*Triticum urartu*	2x	GCA_003073215.1	Ling et al. (2018)
	*Aegilops speltoides* ^g^	2x	SRR949822	
	*Triticum dicoccoides*	4x	GCA_002162155.2	Zhu et al. (2019)

a Species used as outgroup sequence.

b Closest extant relative to maternal progenitor inferred from the plastid genome data (Gordon et al. 2020).

c Closest extant relative to maternal progenitor inferred from the plastid genome data (Cros et al. 1998).

d Closest extant relative to maternal progenitor inferred from the mitochondrial and plastid genome data (Wendel 1989; Chen et al. 2017b).

e Closest extant relative to maternal progenitor inferred from the mitochondrial and plastid genome data (Kolano et al. 2016).

f Closest extant relative to maternal progenitor inferred from the mitochondrial and plastid genome data (Bland et al. 1985; Sasaki et al. 2003).

g Closest extant relative to maternal progenitor inferred from the plastid genome data (Gornicki et al. 2014).

### Orthologous Quintet Inference

Each of the six allopolyploids have subgenomes that are more closely related to those of the sampled diploids than they are to each other. Combined with an outgroup lineage, the resulting tree topology characteristic of allopolyploids (fig. 2) allow for the robust inference of lineage-specific rates of evolution in orthologous quintets. We used a combination of the phylogenetic and syntenic methods to construct orthologous quintets (supplementary fig. S1, Supplementary Material online).

To infer orthologous quintets using the phylogenetic methods, we used Orthofinder v2.2.7 to infer orthologous groups of sequences, termed “orthogroups,” from the whole proteomes (primary transcripts only) of all four species (Emms and Kelly 2019). For each orthogroup, we aligned the CDS sequences in a codon-aware manner using the align_fasta_with_mafft_codon subroutine in the sloan.pm perl module (available at https://github.com/dbsloan/perl_modules) which translates the CDS sequences into amino acid sequences, aligns those amino acid sequences with MAFFT v7.407 (Katoh and Standley 2013), and reverse translates the aligned amino acid positions into the CDS sequences to produce the final alignment. We selected models of molecular evolution for each alignment using jModelTest2 v2.1.10 to identify the model with the highest AICc score (Guindon and Gascuel 2003; Darriba et al. 2012) and inferred phylogenetic trees with the MPI-compatible distribution of PhyML v3.3.20180214 (Guindon and Gascuel 2003). Five random tree starts were performed, and the treespace was further searched using a combination of nearest-neighbor-interchange subtree pruning and regrafting. Support for trees was assessed using 100 bootstrap replicates, and splits with ≤50 bootstrap support were collapsed into polytomies using collapeLowSupportBranches.py (unless otherwise stated, all scripts are available at https://github.com/jsharbrough/allopolyploidCytonuclearEvolutionaryRate/tree/master/scripts).

All monophyletic, minimally inclusive, species-complete subtrees were pruned out of the orthogroup trees using subTreeIterator.py. We next trimmed lineage-specific gene duplicates from subtrees using trimBranches.py, which keeps only the longest sequence or a random sequence in cases where the sequence length is equal across copies. The resulting trimmed subtrees that contained exactly one sequence from each diploid and two sequences from the polyploid represented our set of phylogenetic orthologous quintets. All scripts developed for reading, writing, and manipulating trees are based on the DendroPy package (https://dendropy.org/) (Sukumaran and Holder 2010).

We used the pSONIC (Conover et al. 2021) program to create a genome-wide set of syntenic orthologs. In short, pSONIC employs MCScanX (Wang et al. 2012) to create a list of pairwise syntenic blocks between all the possible pairs of species in each clade, combined with orthogroups identified from the OrthoFinder (Emms and Kelly 2019) to choose which syntenic blocks contained the highest confidence orthologs that were direct descendants of the most recent common ancestor of all species in the clade. Notably, the filtering criteria of collinear groups from our run of pSONIC differed from its formal presentation in that we did not remove collinear groups in which more genes received a “not pass” than “pass” score, and the ends of each collinear block were not trimmed as described in the manuscript describing pSONIC. These developments were made after our analyses were performed with this tool, but before the tool was submitted and reviewed for publication.

To take advantage of both the inference methods, we merged phylogenetic and syntenic orthologous quintets using mergeQuintets.py to produce a high-quality set of quintets that were identical across both methods (i.e., “Intersection”) and a second set of quintets that included all identical quintets plus all the phylogenetic quintets whose members were not present in the syntenic quintets and vice versa (i.e., “Union”). Results from the Intersection dataset (supplementary file S1, figs. S10 and S11, Supplementary Material online) did not differ in any meaningful way from the Union, so only Union results are described in the main text. Phylogenetic quintets that overlapped with but were not identical to syntenic quintets were excluded. Likewise, syntenic quintets that overlapped with but were not identical to phylogenetic quintets were also removed from our final analysis. These conflicting quintets represent a small minority of total quintets and are likely a result of the different methods by which lineage-specific duplicates are handled in the phylogenetic versus syntenic pipelines.

For all non-conflicting orthologous quintets, we realigned the CDS sequences as before and trimmed alignments with Gblocks v0.91b using the codon setting with the -p parameter set to ‘n’ (Castresana 2000). We estimated new models of molecular evolution using the jModelTest2 (Guindon and Gascuel 2003; Darriba et al. 2012) and inferred phylogenetic trees as described above. We tested whether the resulting gene-tree topologies were discordant compared with the overall species tree using the quintetTopology.py script and excluded all quintets from the future analysis that displayed discordant tree topologies (regardless of the bootstrap support). The number and percentage of quintets used in our analyses that exhibited bootstrap values ≥80 in support of the species tree are described in supplementary table S7, Supplementary Material online, and the composition of quintets for each species are described in supplementary tables S8–S13, Supplementary Material online (*Brachypodium*—supplementary table S8, Supplementary Material online, *Chenopodium*—supplementary table S9, Supplementary Material online, *Coffea*—supplementary table S10, Supplementary Material online, *Gossypium*—supplementary table S11, Supplementary Material online, *Nicotiana*—supplementary table S12, Supplementary Material online, *Triticum*—supplementary table S13, Supplementary Material online). There was no difference in any downstream analysis using this higher bootstrap value, so only the data satisfying the lower cutoff are described here.

### CyMIRA-Based Gene Classification

To evaluate the effect of cytonuclear interactions on subgenome-specific evolutionary dynamics, we used a combination of *de novo* targeting predictions and CyMIRA (Forsythe et al. 2019) to partition genes into distinct functional and interaction categories. *De novo* targeting predictions were obtained from four separate targeting prediction programs: iPSORT v0.94 (Bannai et al. 2002), LOCALIZER v1.0.4 (Sperschneider et al. 2017), Predotar 1.03 (Small et al. 2004), and TargetP v1.1b (Emanuelsson et al. 2007). In parallel, we used Orthofinder v2.2.7 to obtain orthology information with the *Arabidopsis thaliana* Araport 11 proteome (Cheng et al. 2017). We combined the *de novo* targeting predictions with the *Arabidopsis*-inclusive orthogroups using the geneClassification.py script. Genes were classified as cytonuclear interacting genes if they shared the same orthogroup as *Arabidopsis* genes, whose products interact with the mitochondrial/plastid genomes or gene products according to the CyMIRA classifications scheme (Forsythe et al. 2019). Genes present in the orthogroups lacking an *Arabidopsis* cytonuclear interacting gene were classified as organelle-targeted if at least one *de novo* prediction tool indicated a mitochondrial or plastid subcellular localization for the gene product and ≥50% of *Arabidopsis* genes present in the orthogroup encode products targeted to the mitochondria or plastids according to CyMIRA. Genes with evidence of dual targeting were included in both the mitochondria-targeted and plastid-targeted data partitions. The resulting genome-wide targeting predictions and the CyMIRA-guided classifications are available at https://github.com/jsharbrough/allopolyploidCytonuclearEvolutionaryRate/tree/master/geneClassification and the pipeline for performing this classification is available at https://github.com/jsharbrough/CyMIRA_gene_classification. The breakdowns of gene functional categories for each genome are provided in table 3, supplementary tables S1 and S2, Supplementary Material online.

We next evaluated whether the retention of genes targeted to the organelles differs across subgenomes by comparing the CyMIRA gene counts across the subgenomes for five out of six polyploid genomes (*N. tabacum* was excluded from this analysis owing to the difficulty in positively identifying subgenomic ancestry for genes lacking a corresponding homoeolog). We performed binomial tests of the NOT genes against the expectations of equal retention, and then, used the *χ*^2^ tests of organelle-targeted gene groups against the genome-wide patterns observed among genes not targeted to the organelles.

### Evolutionary Rate Comparisons

We evaluated the genome-wide signatures of cytonuclear incompatibilities in the organelle-targeted genes using the combination of single-gene and concatenated analyses. For all single-copy quintets whose evolutionary history was consistent with the overall species tree, we removed poorly aligned quintets by estimating the total length of the tree in terms of synonymous substitutions per site (*d_S_*) using model 1 and NSsites = 0 (i.e., branch models) in codeml within PAML v4.9j (Yang 2007). Maximum cutoff values for *d_S_* were determined for each species complex separately and are depicted by red lines in supplementary fig. S9, Supplementary Material online.

After quality filtering, we estimated *d_N_*, *d_S_*, and *ω* for individual quintets using model 1 of the branch models (i.e., NSsites = 0) in codeml as above, and the RateAncestor parameter set to 1. Other PAML parameters included the getSE parameter set to 1, the gamma shape parameter set to a fixed alpha of 0 (i.e., no rate variation among codons), initial omega set to 0.4, and initial kappa set to 2. For each quintet in each functional gene category, we evaluated whether the maternal versus paternal subgenome had a higher *ω* value and a higher *d_N_*. We used the *χ*^2^ tests to evaluate whether individual categories differed from the pattern observed in the group of genes not targeted to the organelles. Using the inferred mutational changes from the RateAncestor output, we also evaluated whether the maternal versus paternal subgenomes had higher numbers of radical amino acid changes (i.e., substitutions between amino acids with substantially different biochemical properties) at sites that were otherwise conserved across the quintet. Substitutions were identified as radical if their score in the CRI matrix (Sharbrough et al. 2018) was >0.5. The accumulation of derived conservative and radical amino acid changes was analyzed in a similar manner to the *ω* and *d_N_* results, using Fisher’s Exact Test to test whether there was a difference compared with genes not targeted to the organelles.

Next, we concatenated quintets according to the gene functional category and estimated *ω* in the maternal versus paternal subgenomes using similar PAML parameters as before. For each PAML run, we repeated the analysis 1,000 times to adequately sample the maximum likelihood plane and found median *ω* values from the replicates for each branch. We then calculated the ratio of paternal to maternal subgenome *ω* values (*ω_PAT_/ω_MAT_*), with a ratio >1.0 indicating faster rates of amino acid sequence evolution in the paternal subgenome and a ratio <1.0 indicating a faster rate of amino acid sequence evolution in the maternal subgenome. We assessed the statistical significance of the degree to which the subgenomes exhibited different rates of amino acid sequence evolution by bootstrapping concatenated alignments at the gene level. For each bootstrap replicate, we randomly sampled genes with replacement from the original concatenation and ran each bootstrapped alignment through five replicate runs of PAML. The median *ω* values of these five replicates were used as the bootstrap replicate values. We then found the ratio of paternal to maternal *ω* values for each bootstrap replicate and functional category to evaluate whether the bootstrapped distributions departed from 1.0. To account for evolutionary forces that are not a result of cytonuclear interactions, we normalized these ratios by dividing by the paternal to maternal *ω* ratio of genes not targeted to either organelle. We inferred two-tailed *P* values directly from the bootstrap distributions. For specific cytonuclear interaction categories, which are composed of only a few dozen genes or less, we manually inspected concatenated alignments, trimmed poorly aligned regions, bootstrapped alignments at the codon level using the python script bootstrapCodons.py, and performed the PAML analyses with a similar approach as before.

Because cytonuclear incompatibilities are only expected when there exists divergence between the two progenitor genomes, we also binned our quintets based on high versus low divergence between diploids for each species and repeated the gene-level bootstrap procedure described above. First, we estimated *d_N_* between diploid relatives for each quintet individually from the gene-specific PAML runs described above and placed the genes according to *d_N_* into two equally sized bins. We then tested whether genes with high levels of amino acid divergence exhibit greater accelerations in *ω* in paternal copies than in genes with lower levels of amino acid sequence divergence. We evaluated statistical significance by bootstrapping alignments at the gene level and comparing the paternal to maternal *ω* ratio distributions from the same gene categories to one another.

## Supplementary Material

msac074_Supplementary_Data

## Data Availability

OrthoFinder results, phylogenetic gene trees with branch lengths, multi-species synteny networks, merged orthologous gene groups, CDS alignments, and analyses of molecular evolution have been made available at https://doi.org/10.6084/m9.figshare.13473207. CyMIRA gene annotations for all species are available at https://github.com/jsharbrough/CyMIRA_gene_classification/tree/master/Species_CyMIRA. All other scripts and trimmed Triticum alignments are available at https://github.com/jsharbrough/allopolyploidCytonuclearEvolutionaryRate. NCBI accession numbers of genomic resources used as part of this study are provided in Table 6.
