# Supplementary material for: Global Patterns of Subgenome Evolution in Organelle-Targeted Genes of Six Allotetraploid Angiosperms
Source: Mol Biol Evol. 2022 Apr 6;39(4):msac074. doi: 10.1093/molbev/msac074 (PMC9040051; doi:10.1093/molbev/msac074)
Supplement: msac074_Supplementary_Data [file msac074_supplementary_data.zip › Supplemental Figures and Figure Legends.pdf]

SUPPLEMENTAL FIGURE LEGENDS

Figure S1. Schematic representation of phylogenetic and syntenic pipelines for inferring orthologous quintets in allopolyploid genomes. The final output of the pipeline was a set of filtered, merged, single-copy quintets, which was used in downstream analyses of rates of protein-sequence evolution.

Figure S2. CyMIRA gene counts in diploid models compared to *Arabidopsis*. The number of genes per category is depicted on the y-axis for both maternal (purple) and paternal (green) diploid models compared to *Arabidopsis* (white). Functional gene categories are listed to the right of each plot: NOT – genes that are non-organelle-targeted, MTNI – mitochondria-targeted non-interacting genes, MTI – mitochondria-targeted interacting genes, MTEC – genes involved in mitochondrial enzyme complexes (subset of MTI), PTNI – plastid-targeted non-interacting genes, PTI – plastid-targeted interacting genes, PTEC – genes involved in plastid enzyme complexes (subset of PTI).

Figure S3. Physical distribution of organelle-targeted genes on chromosomes of focal allopolyploid genomes. Mitochondria-targeted (orange), plastid-targeted (green), and dual-targeted (grey) genes mapped onto chromosomes (black lines) of the six focal allotetraploid genomes. Taxa are arranged from oldest (top) to youngest (bottom), with maternally derived subgenomes on the left and paternally derived subgenomes on the right (excepting tobacco). Chromosome numbers are listed to the left of each chromosome (quinoa chromosomes are numbered according to similarity with *Chenopodium pallidicaule* chromosomes).

Figure S4. CyMIRA gene counts in maternal and paternal subgenomes of allotetraploids relative to diploid models. Bar graph depicting the number of genes present in the maternal subgenome as a proportion of the number of genes present in the maternal diploid model's genome (r_{MAT}) subtracted from the number of genes present in the paternal subgenome as a proportion of the number of genes present in the paternal diploid model's genome (r_{PAT}) for seven functional categories of genes: non-organelle-targeted, mitochondria-targeted non-interacting, mitochondria-targeted-interacting, mitochondria enzyme complexes, plastid-targeted non-interacting, plastid-targeted interacting, plastid enzyme complexes. Polyploid taxa are arranged vertically from oldest (top) to youngest (bottom) for quinoa, cotton, coffee, and *Brachypodium*. Wheat was excluded because the maternal diploid transcriptome from *Aegilops speltoides* was not a good indicator of gene counts.

Figure S5. Rates of protein-sequence evolution in CyMIRA gene categories across the six focal allopolyploids. a) Quintet-wide rates of protein-sequence

evolution across CyMIRA functional categories are depicted in box-and-whisker plots for the combined set of allopolyploids. Data points from each species complex are also shown, the legend for which is provided in the upper right corner of the plot. Boxes are filled according to subcellular compartment, with genes not targeted to the organelles represented by black boxes, genes targeted to either organelle by white boxes, mitochondria-targeted genes are represented by orange boxes, and plastid targeted genes are represented by green boxes. b) Boxplot depicting rates of protein-sequence evolution across allopolyploid species complexes separated by subcellular compartment of localization. Fill color is as described in panel (a).

Figure S6. Poorly aligned regions largely explain elevated *ω* values in paternal homoeologs of wheat mitochondrial enzyme complexes. a) *ω* values from maternal (purple) vs. paternal (green) branches estimated from concatenations of genes that are non-organelle-targeted (left), involved in the mitochondrial enzyme complexes (middle), or involved in plastid enzyme complexes (right) in untrimmed (circles) vs. trimmed (triangles) alignments. The removal of two regions totalling ~240bp accounts for the apparently elevated *ω* values in paternal homoeologs of wheat mitochondrial enzyme complex genes. b-d) Deconstructed *ω* values from concatenated PAML runs for genes (b) non-targeted to the organelles, (c) genes involved in the mitochondrial enzyme complexes, and (d) genes involved in plastid enzyme complexes in untrimmed (top) vs. trimmed (bottom) alignments. Rates of evolution for synonymous (d_S - left) and nonsynonymous (d_N - right) sites are represented by branch lengths, and branches are scaled similarly across functional categories. Green branches represent rates in the paternal subgenome and purple branches represent evolutionary rates in the maternal subgenome of *T. dicoccoides*.

Figure S7. Proportions of genes that have higher *ω* values in paternal vs. maternal copies of organelle-targeted genes. The proportion of genes with higher *ω* values in the paternal homoeolog than in the maternal homoeolog (p_{PAT}) minus the proportion of genes with higher *ω* values in the maternal homoeolog than in the paternal homoeolog (p_{MAT}) is depicted along the x-axis. Mitochondria- (left) and plastid-targeted (right) genes are separated by the degree of interaction: non-interacting genes (top), interacting genes (middle), and genes involved in cytonuclear enzyme complexes (bottom, subset of interacting genes). Proportions are normalized by those found in non-organelle-targeted genes, and genomic bias is denoted by color with maternal bias (i.e., $p_{PAT} – p_{MAT} < 0$) colored purple and paternal bias (i.e., $p_{PAT} – p_{MAT} > 0$) colored green. None of the values exhibited biased proportions according to χ^2 tests, relative to genes not targeted to the organelles. Allopolyploids are arranged from oldest (top) to youngest (bottom) as in Figure 2.

Figure S8. Divergence-binned analysis of subgenomic bias in rates of protein-sequence evolution in six focal allopolyploids. Rates of protein-sequence evolution paternal subgenomes (ω_{PAT}) minus those found in maternal subgenomes (ω_{MAT}) are depicted on the x-axis for non-organelle-targeted genes (left), mitochondria-targeted genes (middle), and plastid-targeted genes (right). Genes were concatenated by functional category and binned according to divergence with high-divergence bins (top) depicted by triangles and low-divergence bins (bottom) depicted by circles. Error bars represent standard errors inferred by PAML. Allopolyploids are arranged from oldest (top) to youngest (bottom). The right two panels are further divided by the degree of intimacy of interaction, with non-interacting genes on top, interacting genes in the middle, and genes that are part of enzyme complexes on bottom. The red-dashed line represents equal rates of protein-sequence evolution in the left panel, but on the right two panels the red-dashed line represents the genome-wide pattern taken from the left panel (i.e., organelle-targeted rates were normalized by non-organelle targeted rates). Maternal bias (i.e.,$\omega_{MAT} > \omega_{PAT}$) occurs left of the red-dashed lines and paternal bias (i.e., $\omega_{PAT} > \omega_{MAT}$) occurs to the right of the red-dashed line.

Figure S9. Alignment filtering based on d_S in orthologous quintets. Individual genes with either total d_S values (wheat, cotton, coffee) or ingroup-only d_S levels (quinoa, tobacco, *Brachypodium*) greater than the cutoff point (indicated by the red line) were excluded from analyses of rates of protein-sequence evolution, as we could not exclude the possibility that those quintets were poorly aligned vs. truly divergent.

Figure S10. Genome-wide bias in ω (d_N/d_S) across maternal and paternal subgenomes, identical quintets only. Log-transformed ratios of ω values in paternal (ω_{PAT}) vs. maternal (ω_{MAT}) subgenomes from concatenations (circles), and underlying bootstrap distributions (density curves) of genes encoding proteins that are not targeted to either the plastids or mitochondria using only quintets that were identical across phylogenetic and syntenic methods. Species panels are arranged vertically from oldest (top) to youngest (bottom). Tobacco was excluded from this analysis because it produced so few syntenic quintets. The red-dashed line indicates equal ω values across subgenomes, left of the red line indicates higher ω values in the maternal subgenomes, and right of the red line indicates higher ω values in the paternal subgenome. Bootstrap distributions of ω ratios that depart significantly ($p < 0.05$) from the red line are filled in solid according to the direction of subgenomic bias (i.e., green: $\omega_{PAT}/\omega_{MAT} > 1.0$; purple: $\omega_{PAT}/\omega_{MAT} < 1.0$; no fill: $\omega_{PAT}/\omega_{MAT} \approx 1.0$).

Figure S11. Ratios of maternal vs. paternal ω values in organelle-targeted genes, identical quintets only. Log-transformed ratios of maternal vs. paternal ω values for

concatenations (black circles) and underlying bootstrap distributions (density curves) of mitochondria- (left) and plastid-targeted (right) genes, including only quintets that were identical across phylogenetic and syntenic methods. Species panels are arranged vertically from oldest (top) to youngest (bottom). Tobacco was excluded from this analysis because it produced so few syntenic quintets. The red-dashed line indicates the $\omega_{PAT}/\omega_{MAT}$ ratio for a concatenation of genes not targeted to the organelles (Figure S9). Ratios left of the red line indicate higher ω values in the maternal subgenome, and ratios right of the red line indicate higher ω values in the paternal subgenome, after accounting for genome-wide patterns. Bootstrap distributions of ω ratios that depart significantly ($p < 0.05$) from the red line are filled in solid according to the direction of subgenomic bias (i.e., green: normalized $\omega_{PAT}/\omega_{MAT} > 1.0$; purple: normalized $\omega_{PAT}/\omega_{MAT} < 1.0$; no fill: normalized $\omega_{PAT}/\omega_{MAT} \approx 1.0$). The intimacy of interactions are indicated on the y-axis from low or no interaction with organelle gene products (top), to interacting genes (middle), to genes involved in mitochondrial or plastid enzyme complexes (bottom).

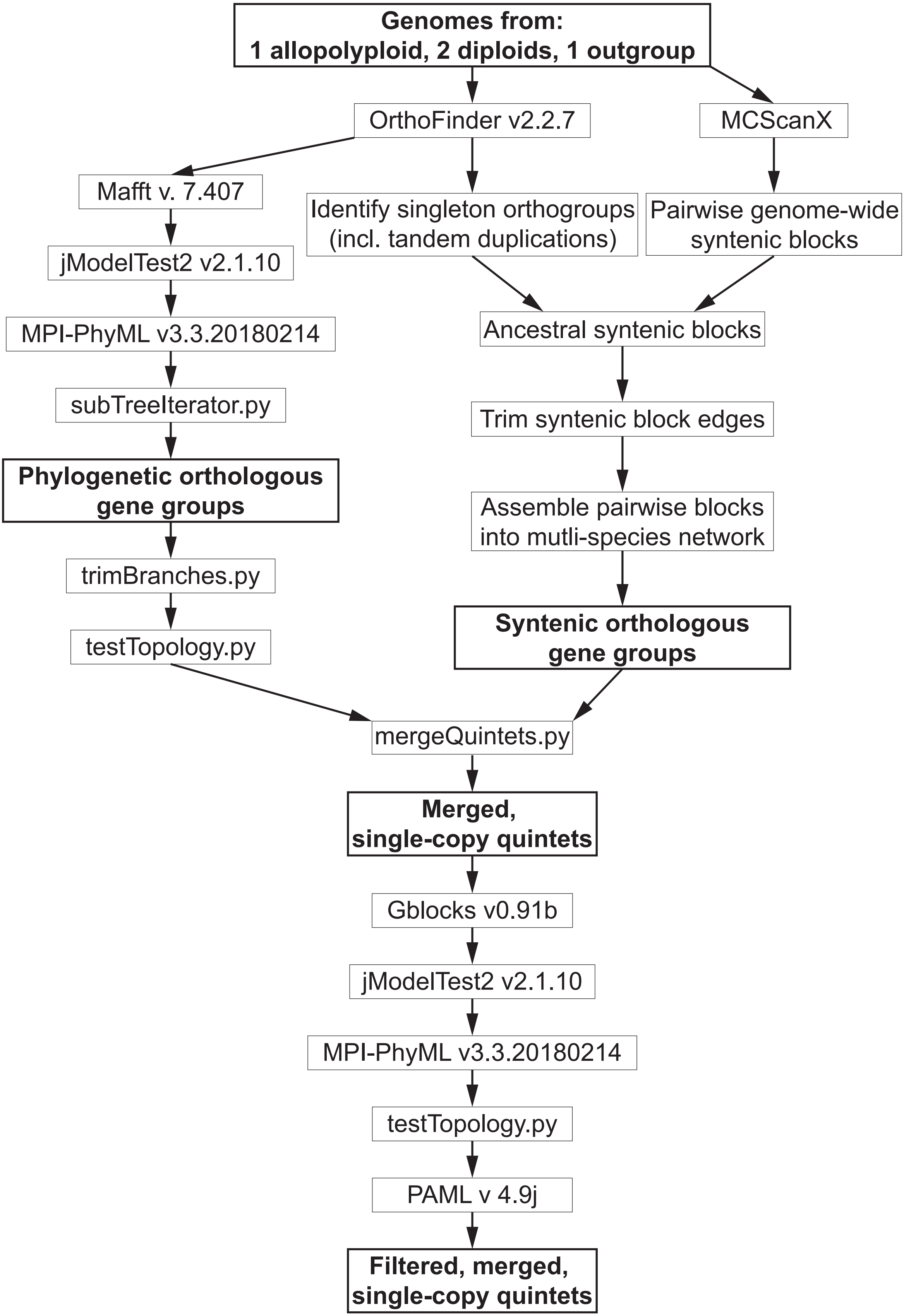
Genomes from:
1 allopolyploid, 2 diploids, 1 outgroup
OrthoFinder v2.2.7
MCScanX
Mafft v. 7.407
Identify singleton orthogroups
(incl. tandem duplications)
Pairwise genome-wide
syntenic blocks
jModelTest2 v2.1.10
MPI-PhyML v3.3.20180214
Ancestral syntenic blocks
subTreeIterator.py
Trim syntenic block edges
Phylogenetic orthologous
gene groups
Assemble pairwise blocks
into mutli-species network
trimBranches.py
testTopology.py
Syntenic orthologous
gene groups
mergeQuintets.py
Merged,
single-copy quintets
Gblocks v0.91b
jModelTest2 v2.1.10
MPI-PhyML v3.3.20180214
testTopology.py
PAML v 4.9j
Filtered, merged,
single-copy quintets

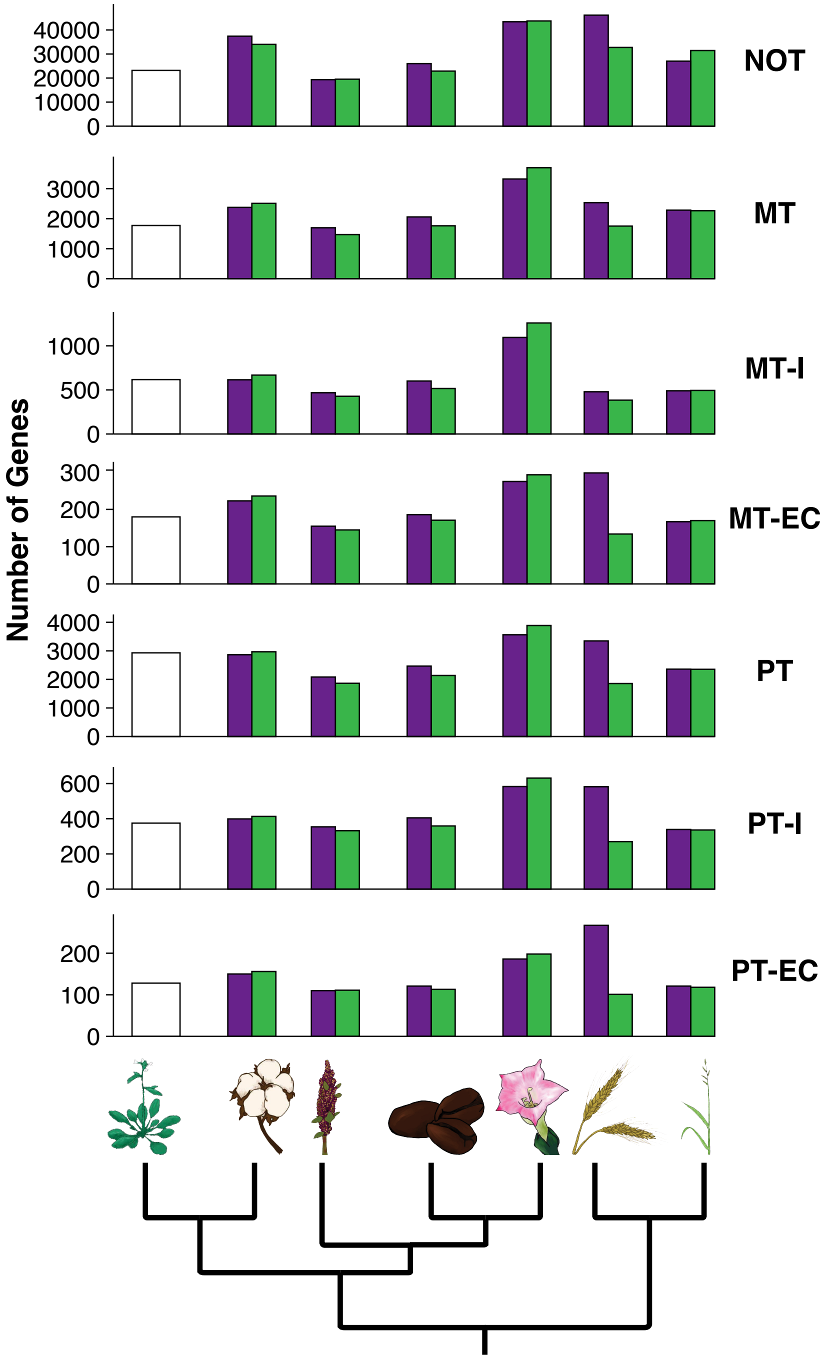
Number of Genes
NOT
MT
MT-I
MT-EC
PT
PT-I
PT-EC
40000
30000
20000
10000
0
3000
2000
1000
1000
500
300
200
100
4000
600
400

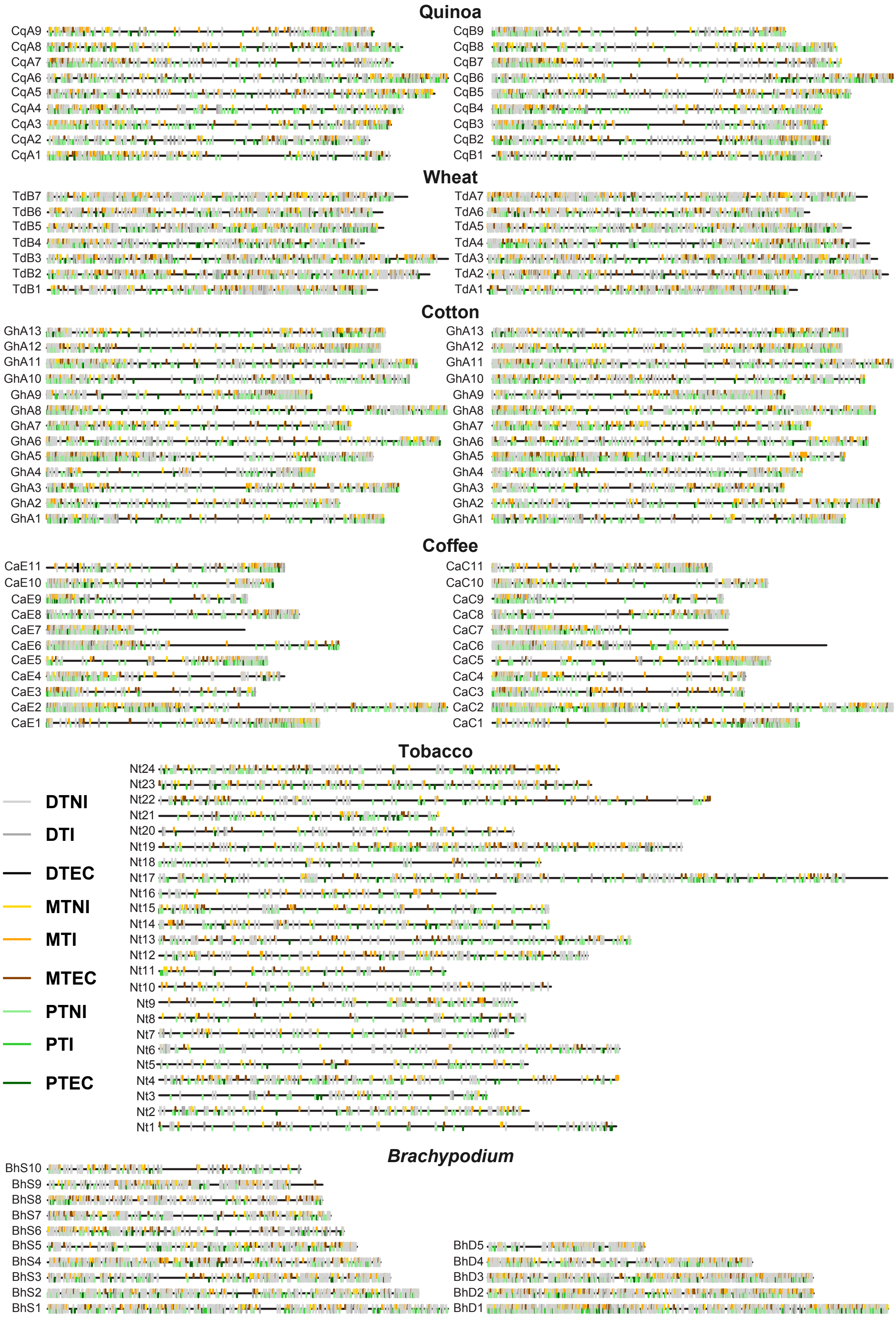

Quinoa
CqA9
CqA8
CqA7
CqA6
CqA5
CqA4
CqA3
CqA2
CqA1
CqB9
CqB8
CqB7
CqB6
CqB5
CqB4
CqB3
CqB2
CqB1
Wheat
TdB7
TdB6
TdB5
TdB4
TdB3
TdB2
TdB1
TdA7
TdA6
TdA5
TdA4
TdA3
TdA2
TdA1
Cotton
GhA13
GhA12
GhA11
GhA10
GhA9
GhA8
GhA7
GhA6
GhA5
GhA4
GhA3
GhA2
GhA1
Coffee
CaE11
CaE10
CaE9
CaE8
CaE7
CaE6
CaE5
CaE4
CaE3
CaE2
CaE1
CaC11
CaC10
CaC9
CaC8
CaC7
CaC6
CaC5
CaC4
CaC3
CaC2
CaC1
Tobacco
Nt24
Nt23
Nt22
Nt21
Nt20
Nt19
Nt18
Nt17
Nt16
Nt15
Nt14
Nt13
Nt12
Nt11
Nt10
Nt9
Nt8
Nt7
Nt6
Nt5
Nt4
Nt3
Nt2
Nt1
DTNI
DTI
DTEC
MTNI
MTI
MTEC
PTNI
PTI
PTEC
Brachypodium
BhS10
BhS9
BhS8
BhS7
BhS6
BhS5
BhS4
BhS3
BhS2
BhS1
BhD5
BhD4
BhD3
BhD2
BhD1

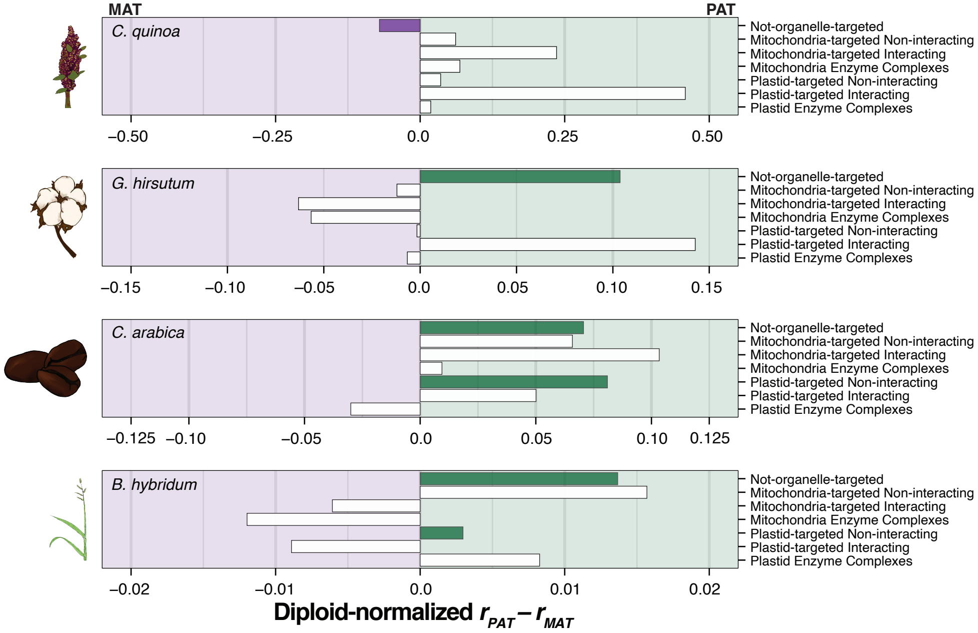

MAT
PAT
C. quinoa
G. hirsutum
C. arabica
B. hybridum
Not-organelle-targeted
Mitochondria-targeted Non-interacting
Mitochondria-targeted Interacting
Mitochondria Enzyme Complexes
Plastid-targeted Non-interacting
Plastid-targeted Interacting
Plastid Enzyme Complexes
–0.50 –0.25 0.0 0.25 0.50
–0.15 –0.10 –0.05 0.0 0.05 0.10 0.15
–0.125 –0.10 –0.05 0.0 0.05 0.10 0.125
–0.02 –0.01 0.0 0.01 0.02
Diploid-normalized $r_{PAT} - r_{MAT}$

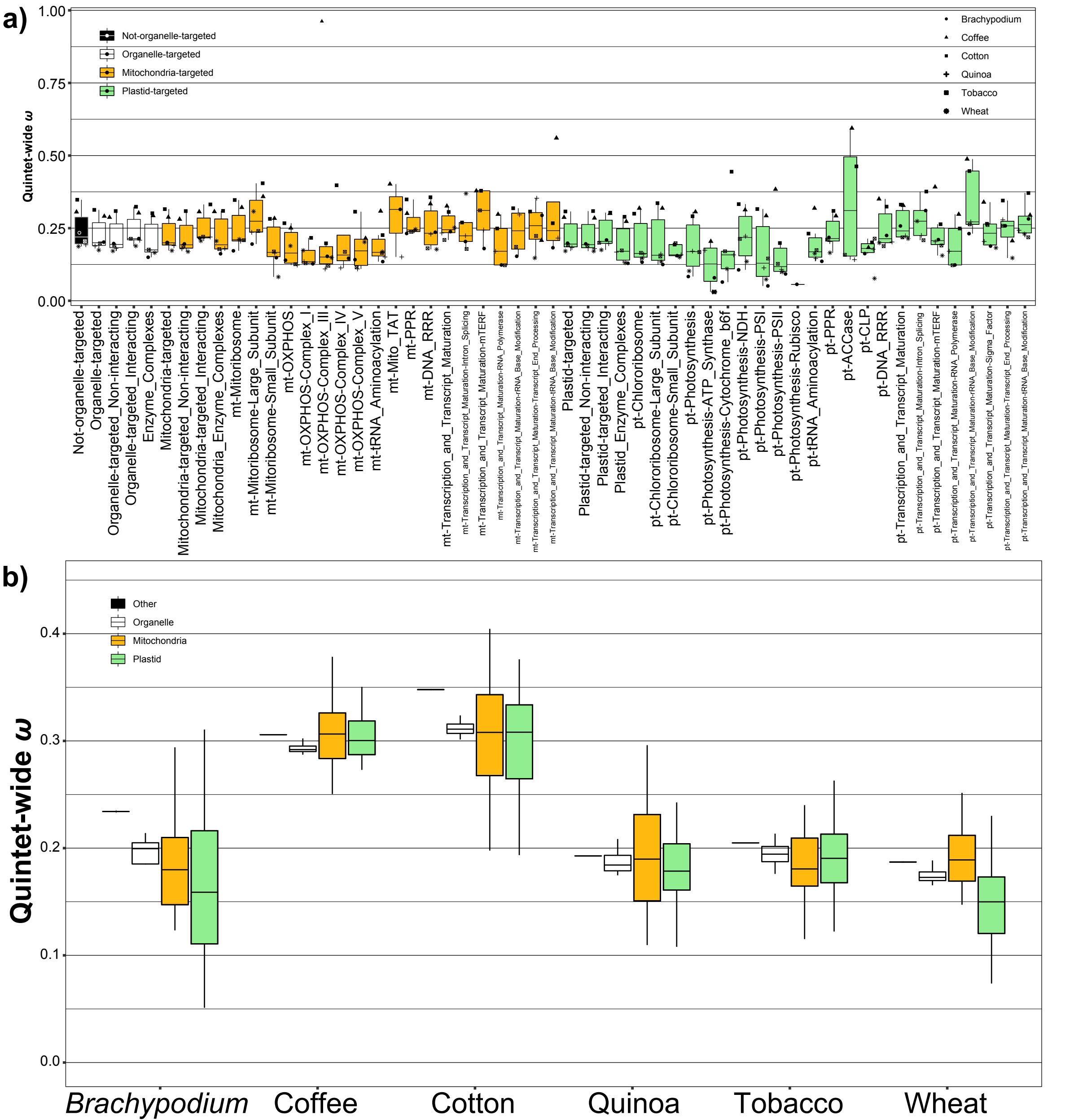
a)
Quintet-wide ω
1.00
0.75
0.50
0.25
0.00
Not-organelle-targeted
Organelle-targeted
Mitochondria-targeted
Plastid-targeted
Brachypodium
Coffee
Cotton
Quinoa
Tobacco
Wheat
Not-organelle-targeted
Organelle-targeted
Organelle-targeted_Non-interacting
Organelle-targeted_Interacting
Enzyme_Complexes
Mitochondria-targeted
Mitochondria-targeted_Non-interacting
Mitochondria-targeted_Interacting
Mitochondria_Enzyme_Complexes
mt-Mitoribosome
mt-Mitoribosome-Large_Subunit
mt-Mitoribosome-Small_Subunit
mt-OXPHOS
mt-OXPHOS-Complex_I
mt-OXPHOS-Complex_III
mt-OXPHOS-Complex_IV
mt-OXPHOS-Complex_V
mt-tRNA_Aminoacylation
mt-Mito_TAT
mt-PPR
mt-DNA_RRR
mt-Transcription_and_Transcript_Maturation
mt-Transcription_and_Transcript_Maturation-Intron_Splicing
mt-Transcription_and_Transcript_Maturation-mTERF
mt-Transcription_and_Transcript_Maturation-RNA_Polymerase
mt-Transcription_and_Transcript_Maturation-rRNA_Base_Modification
mt-Transcription_and_Transcript_Maturation-Transcript_End_Processing
mt-Transcription_and_Transcript_Maturation-tRNA_Base_Modification
Plastid-targeted
Plastid-targeted_Non-interacting
Plastid-targeted_Interacting
Plastid_Enzyme_Complexes
pt-Chlororibosome
pt-Chlororibosome-Large_Subunit
pt-Chlororibosome-Small_Subunit
pt-Photosynthesis
pt-Photosynthesis-ATP_Synthase
pt-Photosynthesis-Cytochrome_b6f
pt-Photosynthesis-NDH
pt-Photosynthesis-PSI
pt-Photosynthesis-PSII
pt-Photosynthesis-Rubisco
pt-tRNA_Aminoacylation
pt-PPR
pt-ACCase
pt-CLP
pt-DNA_RRR
pt-Transcription_and_Transcript_Maturation
pt-Transcription_and_Transcript_Maturation-Intron_Splicing
pt-Transcription_and_Transcript_Maturation-mTERF
pt-Transcription_and_Transcript_Maturation-RNA_Polymerase
pt-Transcription_and_Transcript_Maturation-rRNA_Base_Modification
pt-Transcription_and_Transcript_Maturation-Sigma_Factor
pt-Transcription_and_Transcript_Maturation-Transcript_End_Processing
pt-Transcription_and_Transcript_Maturation-tRNA_Base_Modification
b)
Quintet-wide ω
0.4
0.3
0.2
0.1
0.0
Other
Organelle
Mitochondria
Plastid
Brachypodium
Coffee
Cotton
Quinoa
Tobacco
Wheat

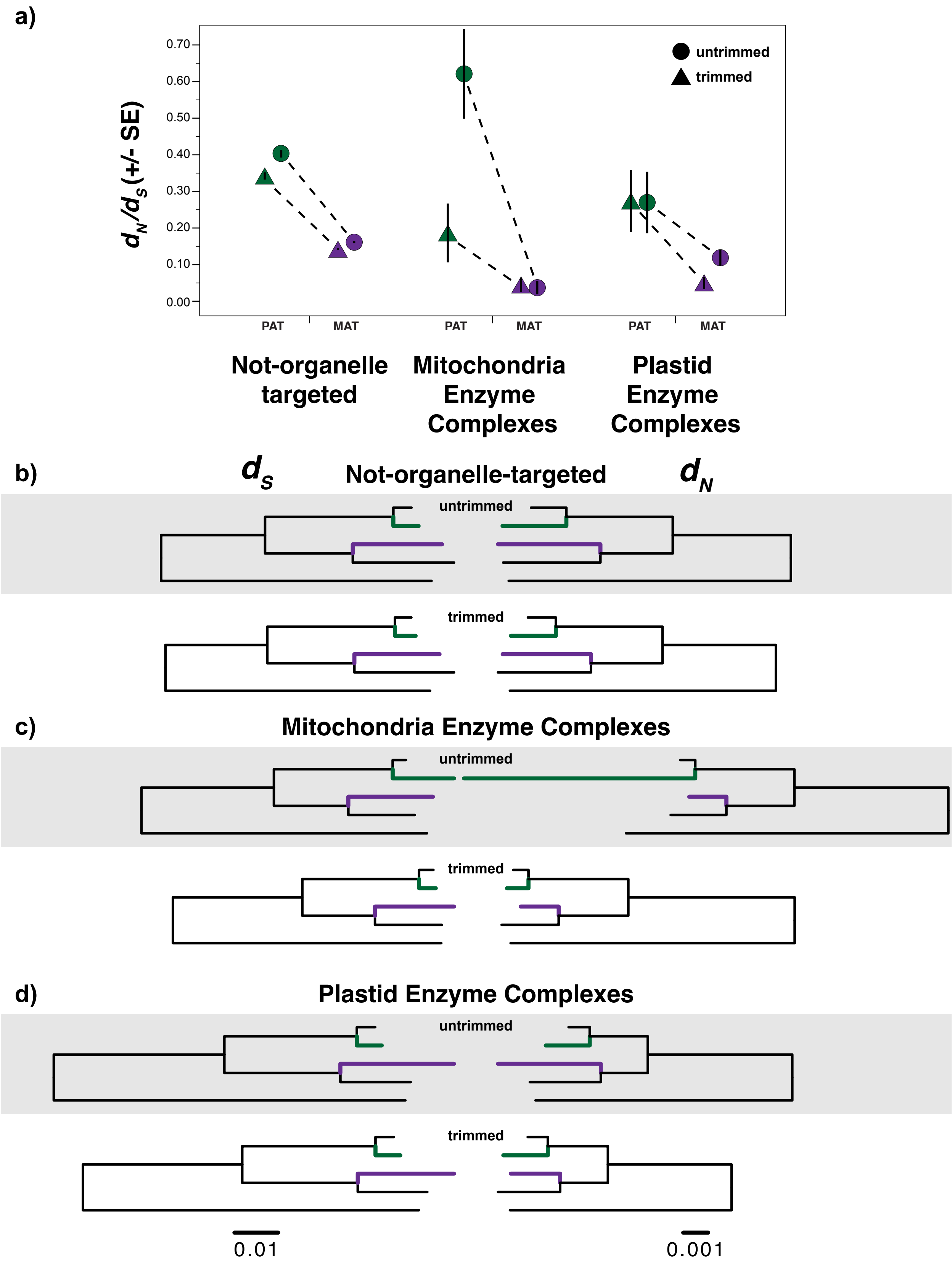

a)
untrimmed
trimmed
d_N/d_S (+/- SE)
0.70
0.60
0.50
0.40
0.30
0.20
0.10
0.00
PAT
MAT
PAT
MAT
PAT
MAT
Not-organelle targeted
Mitochondria Enzyme Complexes
Plastid Enzyme Complexes
b)
d_S
Not-organelle-targeted
d_N
untrimmed
trimmed
c)
Mitochondria Enzyme Complexes
untrimmed
trimmed
d)
Plastid Enzyme Complexes
untrimmed
trimmed
0.01
0.001

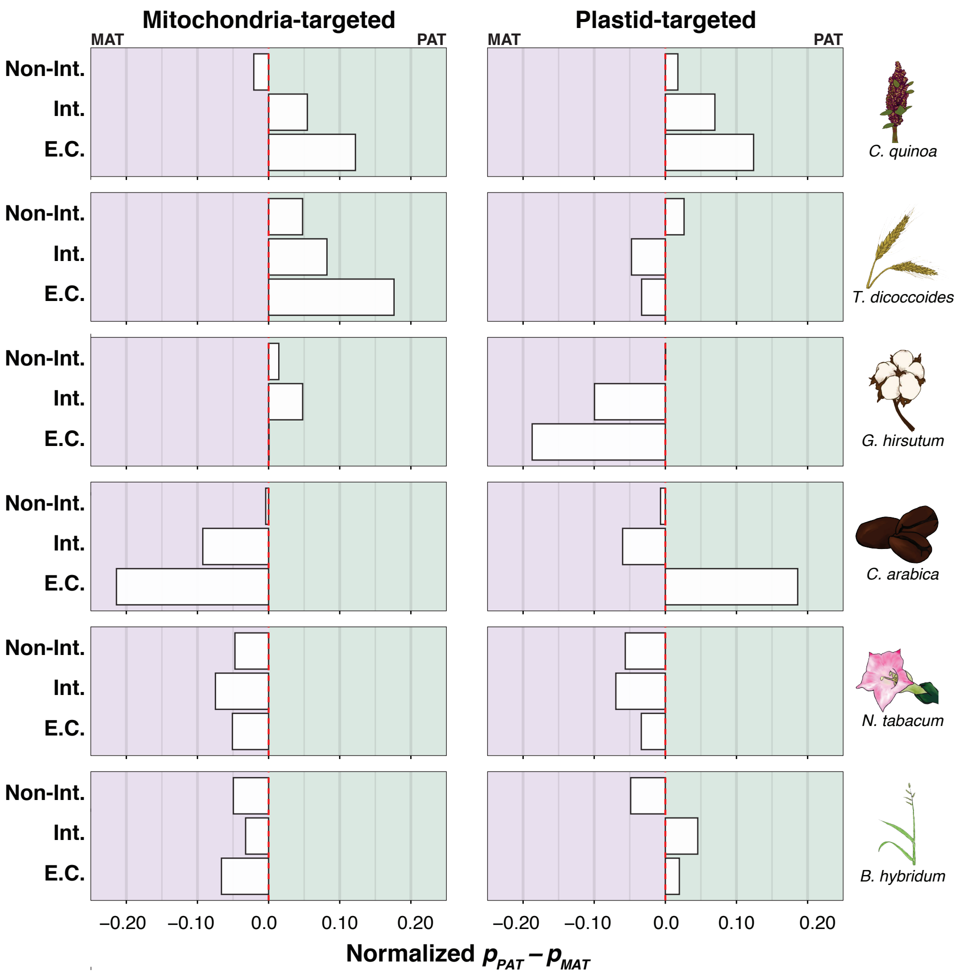

Mitochondria-targeted
Plastid-targeted
MAT
PAT
Non-Int.
Int.
E.C.
C. quinoa
T. dicoccoides
G. hirsutum
C. arabica
N. tabacum
B. hybridum
−0.20
−0.10
0.0
0.10
0.20
Normalized $p_{PAT} - p_{MAT}$

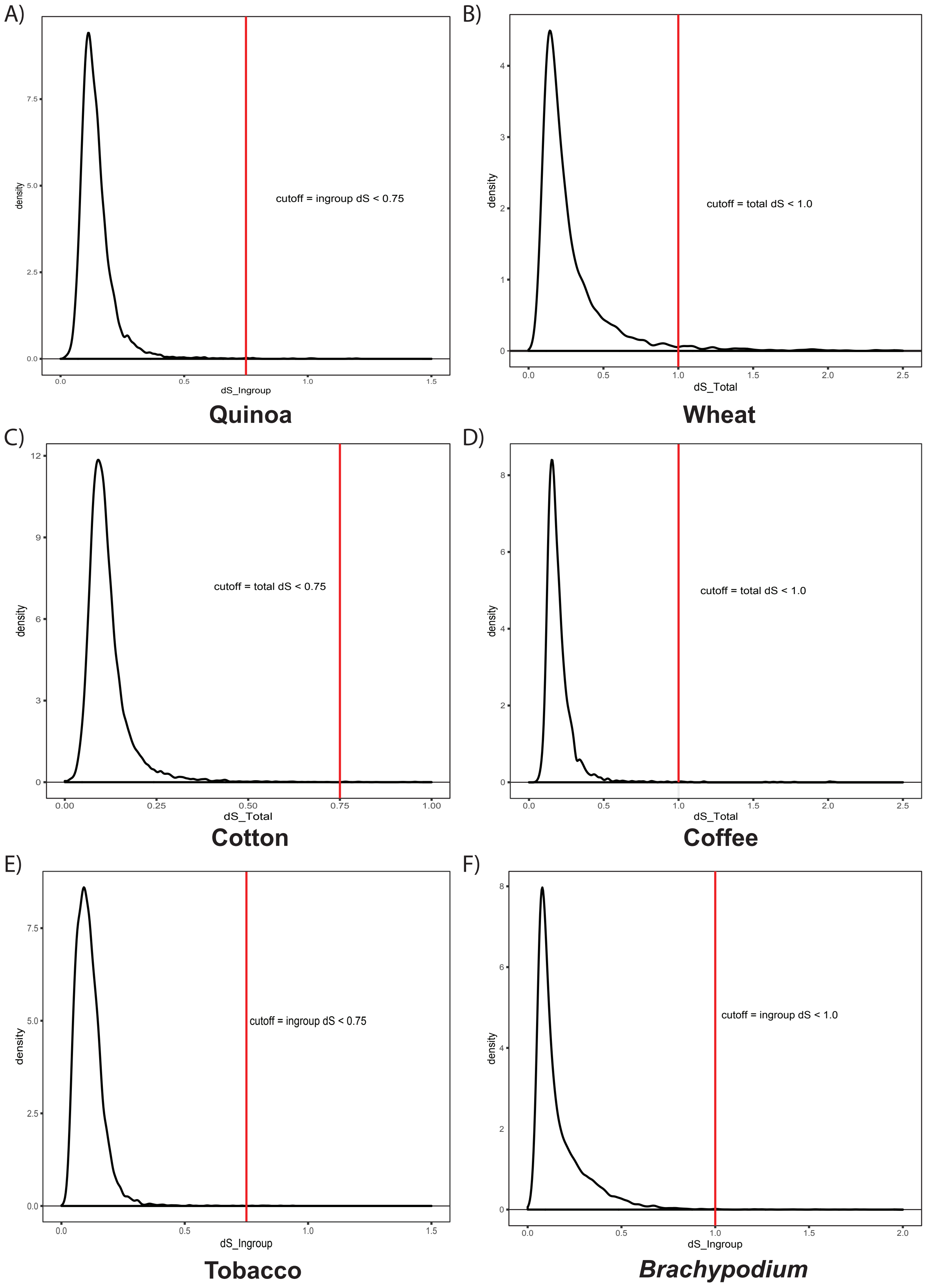
A)
cutoff = ingroup dS < 0.75
density
dS_Ingroup
Quinoa
B)
cutoff = total dS < 1.0
density
dS_Total
Wheat
C)
cutoff = total dS < 0.75
density
dS_Total
Cotton
D)
cutoff = total dS < 1.0
density
dS_Total
Coffee
E)
cutoff = ingroup dS < 0.75
density
dS_Ingroup
Tobacco
F)
cutoff = ingroup dS < 1.0
density
dS_Ingroup
Brachypodium

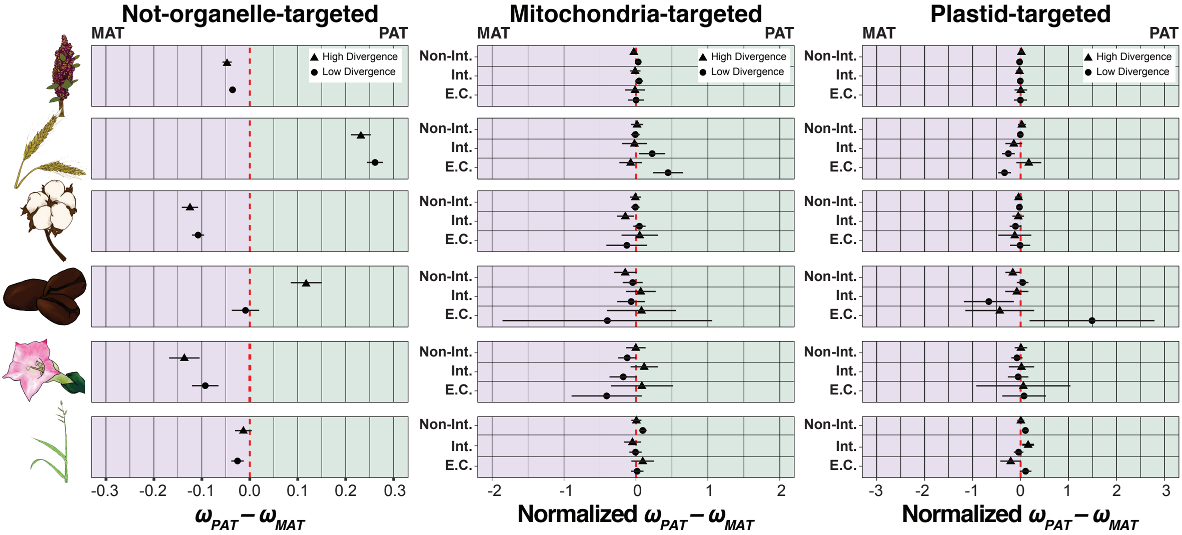

Not-organelle-targeted
Mitochondria-targeted
Plastid-targeted
MAT
PAT
High Divergence
Low Divergence
Non-Int.
Int.
E.C.
-0.3 -0.2 -0.1 0.0 0.1 0.2 0.3
-2 -1 0 1 2
-3 -2 -1 0 1 2 3
$\omega_{PAT} - \omega_{MAT}$
Normalized $\omega_{PAT} - \omega_{MAT}$

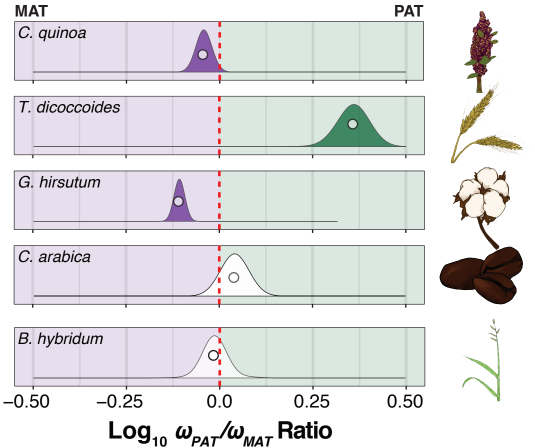
MAT
PAT
C. quinoa
T. dicoccoides
G. hirsutum
C. arabica
B. hybridum
−0.50
−0.25
0.0
0.25
0.50
Log10 ωPAT/ωMAT Ratio

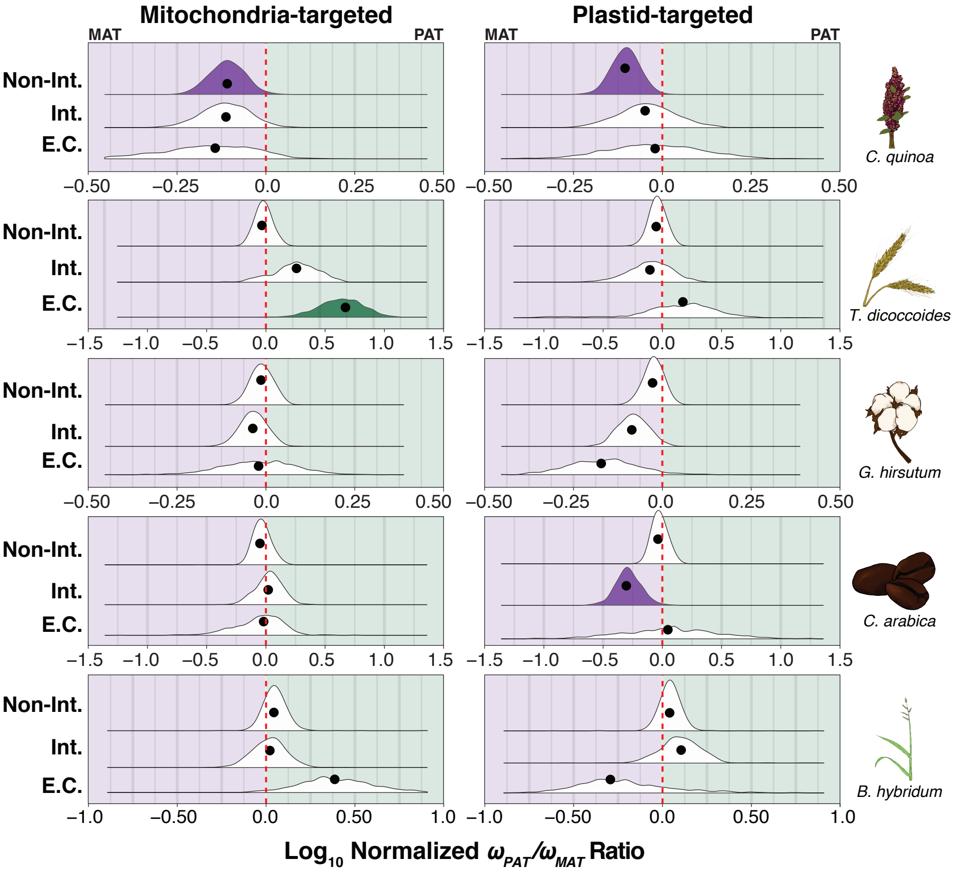

Mitochondria-targeted
Plastid-targeted
MAT
PAT
Non-Int.
Int.
E.C.
C. quinoa
T. dicoccoides
G. hirsutum
C. arabica
B. hybridum
–0.50 –0.25 0.0 0.25 0.50
–1.5 –1.0 –0.5 0.0 0.5 1.0 1.5
–1.0 –0.50 0.0 0.50 1.0
Log10 Normalized ωPAT/ωMAT Ratio
